# Moral parochialism and causal appraisal of transgressive harm in Seoul and Los Angeles

**DOI:** 10.1038/s41598-022-18521-0

**Published:** 2022-08-20

**Authors:** Colin Holbrook, Leehyun Yoon, Daniel M. T. Fessler, Cody Moser, Shairy Jimenez Delgado, Hackjin Kim

**Affiliations:** 1 Department of Cognitive and Information Sciences, University of California, Merced, Merced, CA 95343 USA; 2 Department of Human Ecology, University of California, Davis, Davis, CA 95616 USA; 3 School of Psychology, Korea University, Seoul, 02841 Republic of Korea; 4 Department of Anthropology, University of California, Los Angeles, Los Angeles, CA 90095 USA; 5 Bedari Kindness Institute, University of California, Los Angeles, Los Angeles, CA 90095 USA; 6 Center for Behavior, Evolution, and Culture, University of California, Los Angeles, Los Angeles, CA 90095 USA

**Keywords:** Human behaviour, Biological anthropology

## Abstract

The evolutionary fitness payoffs of moral condemnation are greatest within an individual’s immediate social milieu. Accordingly, insofar as human moral intuitions have been shaped by adaptive design, we can expect transgressive harms to be perceived as more wrong when transpiring in the here and now than when occurring at a distance, or with the approval of local authority figures. This *moral parochialism* hypothesis has been supported by research conducted in diverse societies, but has yet to be tested in an East Asian society, despite prior research indicating that East Asians appraise transgressive acts as being caused by situational and contextual factors to a greater extent than do Westerners, who tend to emphasize dispositional factors (i.e., the transgressor’s personal nature). Here, in a quasi-experiment using field samples recruited in Seoul and Los Angeles, we tested (i) the moral parochialism hypothesis regarding the perceived wrongness of transgressions, as well as (ii) the extent to which these wrongness judgments might be influenced by cross-cultural differences in causal appraisals. Despite notably large differences across the two societies in situational versus dispositional appraisals of the causes of the transgressions, replicating previous findings elsewhere, in both societies we found that transgressions were deemed less wrong when occurring at spatial or temporal remove or with the consent of authorities. These findings add to the understanding of morality as universally focused on local affairs, notwithstanding cultural variation in perceptions of the situational versus dispositional causes of (im)moral acts.

## Introduction

Moral condemnation and punishment of harmful acts appears to be a human universal^1^, functioning to deter antisocial behavior and enhance cooperation in all known human societies^2–4^. Importantly, moral judgments are graded and contingent: some acts seem more wrong than others. Evolutionary functional approaches predict that, due to convergent cost/benefit incentives, transgressive actions occurring in one’s immediate social environment should elicit greater condemnation and related moral sentiments than transgressive acts occurring at a distance. On the one hand, moral condemnation entails a variety of potential costs, such as physical and reputational risks should the target of one’s criticism or their allies respond antagonistically, or the foreclosure of other opportunities due to the investment of one’s time, resources, and motivation in moralizing speech and/or direct punishment of transgressors. On the other hand, moral condemnation and associated punishment of transgressors can convey benefits with regard to one’s reputation by deterring transgressions against oneself or one’s intimates^5^, or by advertising one’s prosocial qualities to potential collaborative partners; in addition, moral punishment may spare one from higher-order punishment (i.e., individuals caught abdicating their obligation to condemn and punish violations may themselves be condemned and punished)^6–8^. Critically, the benefits of moral condemnation accrue primarily within one’s local social milieu, where reputation is known and tracked; absent such benefits, all else being equal, its costs disincentivize engaging in moral condemnation. Taken together, the above considerations provide convergent selective pressures, both at the level of the individual, and with regard to cultural group selection of moral norms^9^, for moral evaluation to take spatial or temporal distance into account such that remote misdeeds arouse a relatively muted level of condemnation.

In addition to relative spatiotemporal distance, the pronouncements of authority figures should similarly influence moral evaluation^10^. The functional benefits of moral condemnation only accrue when in-group members share perceptions of acts as objectionable. As norms change over time, individuals are incentivized to update their moral sentiments to align with those of their communities. Prestigious local figures are often the arbiters of normative standards; attending to their moral evaluations can guide individuals in tracking shifts in moral norms. Beyond the aforementioned incentive to entrain one’s moral sentiments with those of one’s community in order to reap the benefits of moral condemnation, there are also parallel incentives to avoid the costs related to refraining from condemning actions that high-status local authorities deem morally wrong (e.g., the costs of being targeted for third-party punishment, including reputational consequences such as exclusion from opportunities to cooperate), as well as to avoid the costs of condemning actions that high-status local authorities deem morally acceptable or obligatory (e.g., the consequences of signaling antagonism toward, or unwillingness to accede to, authorities).

In summary, the *moral parochialism* hypothesis outlined above predicts that transgressive harms will be condemned with greater intensity when committed in one’s local community than when occurring far away, long ago, or with the consent of local authorities^9^. To the extent that the functional cost/benefit logic applies across human societies, moral parochialism should be universal. To date, the moral parochialism hypothesis has been tested in seven societies, including two large-scale societies (the urban communities of San Jose and Santa Monica in California, and Storozhnitsa, a town in Western Ukraine) and five small-scale societies: indigenous egalitarian South American horticulturalists (a Tsimane’ community in Bolivia and a Shuar community in Ecuador), a clan-based indigenous Polynesian community (Yasawa Island, Fiji), a rural Indonesian community of rice agriculturalists (Karo Batak), and a clan-based community of Melanesian horticulturalists (Sursurunga)^10,11^. Participants in all seven societies judged actions involving unjust harms to be less morally wrong when framed as having occurred either in a faraway land or long ago; the hypothetical consent of local authorities similarly diminished wrongness ratings in four of the societies, with the remaining three societies evincing similar but non-significant trends. Although it is notable that the pattern observed in such a diverse array of societies accorded with predictions, the moral parochialism hypothesis has yet to be tested in an East Asian society, a gap which appears particularly important given prior research indicating that members of East Asian societies differ substantively from members of Western societies in their perceptions of the causes of transgressive harms.

Researchers have studied cultural differences in appraisals of the causes of social behavior for decades, primarily with regard to the tendency to over-attribute behavior to dispositional, personality-oriented factors rather than situational explanations, a bias termed the *fundamental attribution error*^12–14^. Individuals from East Asian societies have been found to be less susceptible to the fundamental attribution error than North Americans, as the former tend to consider contextual factors as contributing to behavioral outcomes to a greater extent^15–17^. Scholars have posited that, correspondent to parallel differences in collectivism versus individualism, East Asian cultures holistically construe human behavior as caused by complex interactions between personal and situational factors relative to North American cultures, which are characterized by dispositional causal models^13,18,19^.

The East/West difference in styles of causal attribution appears to extend to evaluations of the causes of harmful transgressions. Morris and Peng compared articles written about murders in the leading Chinese-language newspaper (World Journal) to articles written about closely comparable murders in The New York Times^20^. Chinese reporters emphasized contextual factors (e.g., the murderer’s isolation from his community, his poor relationship with the victim, or the availability of firearms) to a significantly greater extent than did U.S. reporters, who emphasized dispositional factors (e.g., the murderer’s aggressive personality). They observed a similar pattern in a convergent study comparing the appraisals of Chinese versus U.S. participants tasked with evaluating the causes of murder cases. Choi and colleagues likewise found that South Korean participants endorsed a more holistic style of causal appraisal of social behavior than U.S. participants, who endorsed a more dispositional style, and that U.S. participants cited dispositional factors as causing murder to a significantly greater extent than did Korean participants^21^.

In the present study, we integrated the literature on East/West cultural differences in causal appraisal of transgressive harm with the first test of the moral parochialism hypothesis in an East Asian society. We compared moral evaluations of the same transgressive harms in a cross-cultural study conducted in South Korea and in the U.S. We hypothesized that:

- As in previous cross-cultural research^10^, both Korean and U.S. participants would rate transgressive harms as more wrong when occurring in their home communities than when occurring
  - at a spatial distance in another society,
  - at a temporal distance,
  - or with the consent of local authorities.
- We expected baseline wrongness ratings to positively correlate with ratings of the reputational consequences for the transgressor as a bad person. (Essentially, this correlation functions as a manipulation check to confirm that the actions described were perceived as morally transgressive.)
- We anticipated that Korean participants would attribute transgressive harms to situational rather than dispositional factors to a greater extent than would U.S. participants.

In addition to these directional predictions, the present design provided an opportunity to explore

- associations between appraisals of wrongness and appraisals of cause,
- associations between appraisals of cause and appraisals of reputational consequences,
- and potential differences between societies with regard to wrongness evaluations.

With regard to potential effects of society on wrongness evaluations, we tentatively anticipated that the Korean sample might evince a more pronounced degree of moral parochialism (i.e., reductions related to the context in which transgressions occur), given that East Asian societies understand social behavior in a more contextually situated and relational fashion relative to Western societies. These predictions and exploratory questions were tested in a quasi-experiment with field samples recruited in the urban settings of Seoul and Los Angeles.

## Methods

The full materials and data are available in the Supplementary Information (SI) and on the Open Science Framework: https://osf.io/jq2vm/. The study hypotheses, analysis plan, and target sample sizes were not pre-registered.

### Participants

52 Korean and 75 U.S. participants were recruited on the streets of Seoul and Los Angeles in exchange for 10,000 KRW or 10 USD in compensation, respectively (a comparable sum at the time of data collection, the summer of 2016). Recruitment aimed to meet or exceed the sample sizes used in the seven field sites (*N*s 30–49) reported in Fessler et al.^10^ during the period available for data collection, leading to roughly comparable but uneven sample sizes in the two societies, as data collection in Los Angeles proved to be more successful. Data were pre-screened for completeness and according to concerns noted by the research assistants; four U.S. participants did not complete the study (one of whom also wrote jokes on the demographics page), one U.S. participant was interrupted by a friend, and one U.S. participant reported that they personally recognized the research assistant, raising reputation management concerns. This left a final sample of 52 Korean participants (42.3% female) ranging in age from 20 to 58 (M = 34.13, SD = 10.88) and 69 U.S. participants (52.2% female) ranging in age from 18 to 60 (M = 31.41, SD = 12.24). (Follow-up analyses confirm that the same overall pattern of results is also obtained if including the raw, unfiltered sample.) Although our target sample sizes were based on the goal of meeting or exceeding the size of the samples reported in^10^, power analyses were also later conducted using G*Power (version 3.1.9.6^22^) to determine the minimum sample sizes required to test (i) the moral parochialism hypothesis, and (ii) the effect of society on appraisals of the causes of transgressions, assuming medium effects. Results indicated that the required sample sizes to achieve 80% power, at a significance criterion of *α* = 0.05, were *N* = 24 (within each society) using a repeated-measures ANOVA for tests of the moral parochialism hypothesis, and *N* = 126 to test the effect of society on causal appraisals using a between-subjects ANOVA. Accordingly, the present sample appears to have been adequately powered for detecting medium effects, and more than adequate for detecting large effects.

The study materials were translated from English to Korean by co-author LY, then back-translated independently by a fluently bilingual research assistant who was not otherwise involved in this research (see SI for the full materials and the back-translated protocol). The study was approved by the University of California, Los Angeles, Institutional Review Board, informed consent was obtained prior to participation, and all methods were in accord with relevant guidelines and regulations.

### Moral parochialism

The first section of the study closely replicated the structured-interview design employed by Fessler and colleagues in their cross-cultural study of moral parochialism^10^. In a within-subjects design, participants listened to and evaluated six transgression scenarios involving unambiguously unjust harm: a man cheating a stranger in a market transaction (‘Market Cheating’); a man battering his wife without provocation (‘Domestic Battery’); a man striking a friend after the friend accidentally injured him (‘Unintentional Harm’); a man stealing a stranger’s money (‘Stranger Theft’); a man knowingly spreading a false rumor that another man is a thief (‘Defamation’); the initiator of a fight bribing a witness to lie about who was at fault, resulting in the innocent party being punished (‘Injustice’) (see SI). (A seventh scenario concerning sexual assault used in previous research was omitted from this design as potentially evoking unnecessary distress.) For each scenario, the research assistant read the scenario aloud, confirmed that the participant comprehended the events and individuals involved, then asked the participants to report their appraisals. First, participants rated the Baseline severity of the wrongness of the transgression (“In your personal view, how good or bad is what [NAME] did?”; 1 = *Extremely Bad*; 5 = *Neither Good nor Bad*; 9 = *Extremely Good*; hashmarks rather than numerals were used to demarcate each point). Following the wrongness rating, the reputational consequences for the transgressor were estimated, using the same scale to report the extent to which people in the transgressor’s community would think they were “a good person or a bad person”. Next, three context conditions were presented and the wrongness of the transgression was once again evaluated using the same scale: (i) local community leaders state that this type of action was ‘not bad’ (*Authority Consent*); (ii) the action occurs in the distant past (*Temporal Distance*); and (iii) the action occurs in a distant society (*Spatial Distance*). The scenarios were presented in one of four orders (randomly assigned). Likewise, although the baseline wrongness and reputational consequence questions were always presented first and in fixed order, the three post-baseline context questions varied across the four orders (see SI). For each scenario, the research assistant confirmed that the participant comprehended the scenario before proceeding to evaluations of the severity of the transgression. The wrongness ratings were averaged across the six scenarios to create composite ratings for each of the four contexts (*α*s 0.80–0.89); the reputational consequence ratings were likewise averaged to create a composite (*α* = 0.68).

Following the baseline wrongness rating, participants were asked to provide a justification. These qualitative responses are not analyzed here, but generally consisted of restatements of the transgression (e.g., “because it is stealing”) or assertions of wrongness without further explanation (e.g., “that is bad”); the full justifications are available in the archived dataset.

### Causal attribution

The second section of the study focused on appraisals of the causes of the transgressive acts. Participants were once again presented the six scenarios in the Baseline context (in one of four orders, see SI), and asked to evaluate the cause of each transgressive act, according to two questions, presented in counterbalanced order: “How much do you feel that other people would have been likely to [PERFORM TRANSGRESSIVE ACT] if they found themselves in the exact same situation?”; 1 = *Almost No one*; 9 = *Almost Anyone* (the *Others Would Also Transgress* item); “How much do you feel that [TRANSGRESSIVE ACT OCCURED] because of the kind of person that [NAME] is, in comparison to all of the other causes?” 1 = *Completely Caused by the Kind of Person He Is*; 9 = *Completely Caused by the Situation* (the *Caused by Situation* item). These causal attribution ratings were averaged across the six scenarios to create composite ratings (*α*s > 0.86). As intended, the composites of the two causal attribution questions were correlated in both the U.S sample, *r*(68) = 0.30, *p* = 0.012, and the Korean sample, *r*(51) = 0.35, *p* = 0.011, but only to a moderate extent, indicating that the two questions assessed dispositional versus situational causal appraisals in complementary, non-redundant ways.

Finally, participants were either verbally asked demographic questions (in the Seoul sample) or handed a survey page and a pen with which to answer demographic questions (in the U.S. sample) before being thanked, paid, and debriefed.

### Statistical methods

The data were analyzed using SPSS 27.0 software^23^. The effects of society sampled (Los Angeles, Seoul) and of transgression context (Baseline, Authority Consent, Spatial Distance, Temporal Distance) on ratings of wrongness were analyzed using a mixed-measures analysis of variance (ANOVA), with society as the between-subjects variable and context as the within-subjects variable. The wrongness ratings were composited across the six transgressions (for parallel analyses of effects of society or context for the individual scenarios, see SI). Ratings of the causes of the six transgressions were likewise composited, with effects of society on composite causal appraisals assessed using a between-subjects ANOVA (for parallel analyses of effects of society on the individual scenarios, see SI). Effect sizes were reported as partial eta squared values. Associations between wrongness, causal appraisals, and reputation ratings were assessed according to Pearson’s correlations. We did not adjust for multiple comparisons (e.g., using Bonferroni corrections).

## Results

Preliminary tests revealed no effects of sex, nor interactions between sex and society, on either wrongness or causal attribution ratings. Accordingly, sex was dropped from the analyses.

### Perceived wrongness and reputational consequences

Pooling societies, as anticipated, ratings of composite baseline wrongness and reputational consequences were positively correlated, *r*(120) = 0.64, *p* < 0.001, and follow-up tests confirmed that this association obtained in both the Seoul sample, *r*(51) = 0.49 *p* < 0.001, and the Los Angeles sample, *r*(68) = 0.74, *p* < 0.001. Of the six individual scenarios, we observed one between-society difference in Baseline wrongness ratings (the Unintentional Harm scenario, Seoul less wrong than Los Angeles, see SI Table S3), and one between-society difference in ratings of reputational consequences (the Domestic Battery scenario, Seoul less wrong than Los Angeles, see SI Table S11), suggesting that, notwithstanding likely cultural differences in meaning and normative severity, overall, the actions were understood to be transgressive in both Seoul and Los Angeles.

### Effects of society and context on perceived wrongness

We conducted a 2 (society) by 4 (transgression context) mixed-model ANOVA. As hypothesized, there was a main effect of transgression context, *F*(3, 357) = 22.61, *p* < 0.001, *η*_*p*_^2^ = 0.16, with the composite transgressions rated significantly more wrong in the Baseline condition than in the contexts of Authority Consent (*p* < 0.001, *η*_*p*_^2^ = 0.18), Spatial Distance (*p* < 0.001, *η*_*p*_^2^ = 0.28), or Temporal Distance (*p* < 0.001, *η*_*p*_^2^ = 0.31). This main effect was qualified by two significant interaction effects between society and context condition, wherein the Seoul participants rated the composited transgressions as less wrong relative to baseline than did Los Angeles participants in the context of Spatial Distance, *F*(1, 119) = 7.51, *p* = 0.007, *η*_*p*_^2^ = 0.06, and in the context of Temporal Distance, *F*(1, 119) = 5.35, *p* = 0.022, *η*_*p*_^2^ = 0.04. There was no such interaction with regard to the context of Authority Consent, *p* = 0.611. Both societies rated the transgressions as significantly less wrong relative to baseline for all three contexts (see Table 1).

**Table 1:** Transgression context and composite wrongness ratings.

Context	M	SD	*p*	*η*_*p*_^2^
**Los Angeles (*N* = 69)**				
Baseline	1.98	0.68		
Authority consent	2.43	1.15	< 0.001	0.19
Spatial distance	2.23	0.95	< 0.001	0.18
Temporal distance	2.31	0.95	< 0.001	0.28
**Seoul (*N* = 52)**				
Baseline	2.01	0.49		
Authority consent	2.38	0.81	0.001	0.21
Spatial distance	2.61	0.99	< 0.001	0.34
Temporal distance	2.64	1.05	< 0.001	0.34

Lower ratings indicate appraisals of the transgressive act as more wrong. *P* values and effect sizes refer to planned within-subjects contrasts using Baseline context ratings as the reference condition.

There was also a main effect of society such that the Seoul sample rated the transgressions as less wrong on average in the context of Spatial Distance, *F*(1, 119) = 4.42, *p* = 0.038, *η*_*p*_^2^ = 0.04, 95% CI [0.02, 0.73], with a similar nonsignificant trend in the Temporal Distance context, *F*(1, 119) = 3.26, *p* = 0.074, *η*_*p*_^2^ = 0.03, 95% CI [− 0.03, 0.69], relative to the Los Angeles sample (see Table 1). There were no such effects of society on baseline wrongness ratings, *p* = 0.806, or wrongness within the context of Authority Consent, *p* = 0.775.

Follow-up analyses revealed that the same overall pattern obtained within the individual transgression scenarios (see SI Tables S1–S6). Transgressions were rated as significantly more wrong at baseline than when either spatially or temporally distant for all six scenarios in both societies, with one exception: there was no effect of spatial distance for the 'defamation' scenario in the Los Angeles sample. Although authority consent also significantly reduced wrongness ratings in many cases, this effect was not observed in the ‘injustice’, ‘unintentional harm’, or ‘defamation’ scenarios in the Seoul sample, nor in the ‘domestic battery’ scenario in the Los Angeles sample. These exceptions, while departing from predictions, demonstrate that participants evaluated the scenarios and contexts in a way which cannot be explained by demand characteristics (i.e., inferring that they were ‘supposed’ to attenuate their wrongness ratings because they were asked repeatedly). The variation within both samples in the relative magnitude of the shifts in wrongness ratings in each context also indicates that participants’ ratings reflect their moral sentiments rather than demand effects.

### Effects of society on causal attribution

**Figure 1:**
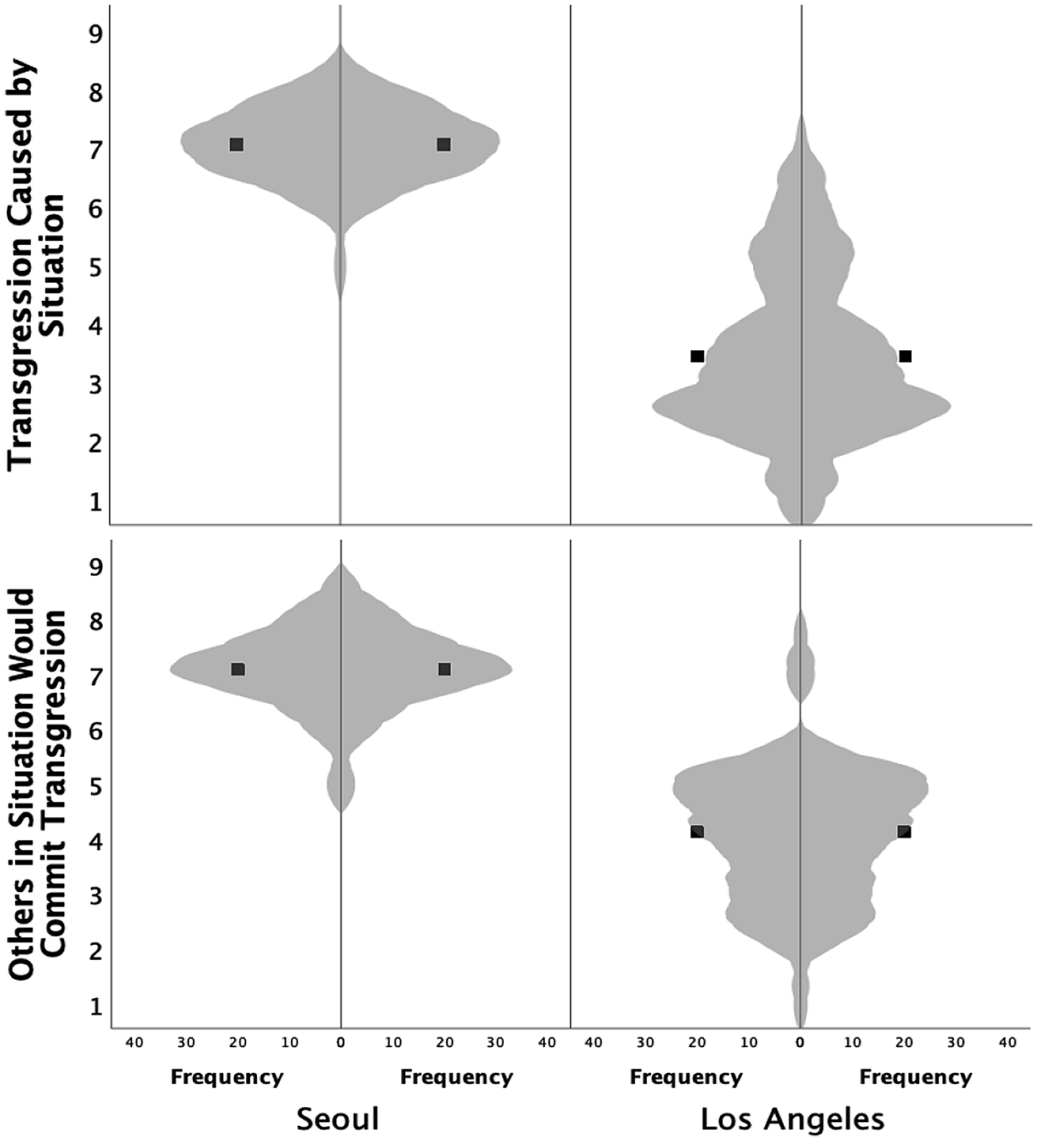
Between-society differences in causal attribution, averaging across transgression scenarios. Relative to participants in Los Angeles, participants in Seoul appraised transgressions as caused by the situational circumstances moreso than the personal disposition of the transgressor (top), and more likely to have been committed regardless of the particular person present in the same situation (bottom). The violin plot outlines illustrate kernel probability density; the width of the shaded area represents the proportion of data located there and the black squares indicate the means (see text for details).

Between-subjects ANOVAs revealed that, as hypothesized, participants in the Los Angeles sample rated the cause of the composite transgression as significantly more attributable to the perpetrator relative to participants in the Seoul sample, who tended to holistically attribute the cause of the transgression to the situation. This societal difference was evidenced using both measures intended to assess causal attribution, in notably large effects. To a greater degree than Los Angeles participants, Seoul participants indicated that most people under the same circumstances would have transgressed (i.e., the Others Would Also Transgress question) (*Los Angeles*: *M* = 4.13, *SD* = 1.27; *Seoul*: *M* = 7.09, *SD* = 0.74), 95% CI [2.57, 3.35], *F*(1, 119) = 226.06, *p* < 0.001, *η*_*p*_^2^ = 0.66, and that the transgression was caused by situation moreso than the personal qualities of the transgressor (i.e., the Caused by the Situation question) (*Los Angeles*: *M* = 3.40, *SD* = 1.42; *Seoul*: *M* = 7.06, *SD* = 0.62), 95% CI [3.24, 4.08], *F*(1, 119) = 301.62, *p* < 0.001, *η*_*p*_^2^ = 0.72 (see Fig. 1). Follow-up tests revealed that the same pattern obtained for all six scenarios (see Table 2).

**Table 2:** Appraisals of the causes of transgressive harms by scenario and society.

Scenario	Seoul *N* = 52 M (SD)	Los Angeles *N* = 69 M (SD)	*F*	*p*	*η*_*p*_^2^	95% CIs
**Stranger theft**						
Caused by situation	7.24 (1.33)	3.57 (2.18)	115.35	< 0.001	0.49	3.00, 4.35
Others would also	7.33 (1.48)	4.49 (2.10)	69.02	< 0.001	0.37	2.16, 3.51
**Domestic battery**						
Caused by situation	6.85 (1.09)	2.48 (2.06)	193.46	< 0.001	0.62	3.75, 4.99
Others would also	7.04 (1.08)	2.52 (1.65)	293.66	< 0.001	0.71	4.00, 5.04
**Unintentional harm**						
Caused by situation	7.12 (1.50)	4.06 (2.57)	58.58	< 0.001	0.33	2.27, 3.85
Others would also	6.74 (1.64)	4.15 (2.01)	57.80	< 0.001	0.33	1.92, 3.27
**Market cheating**						
Caused by situation	6.86 (1.36)	2.88 (2.19)	132.77	< 0.001	0.53	3.29, 4.65
Others would also	7.33 (1.38)	3.80 (2.08)	111.99	< 0.001	0.49	2.87, 4.19
**Defamation**						
Caused by situation	7.22 (1.53)	3.55 (2.36)	95.50	< 0.001	0.45	2.93, 4.41
Others would also	7.08 (1.41)	4.75 (2.19)	44.45	< 0.001	0.27	1.63, 3.01
**Injustice**						
Caused by situation	7.09 (1.43)	3.87 (2.31)	78.42	< 0.001	0.40	2.50, 3.94
Others would also	7.03 (1.46)	5.06 (1.95)	37.35	< 0.001	0.24	1.33, 2.61

Lower ratings indicate appraisals of the transgressive act as caused by dispositional factors; higher ratings indicate appraisals of the transgressive act as caused by situational factors. *P* values, effect sizes, and 95% CIs reflect contrasts between the two societies.

#### Society, causal attribution and perceived wrongness

There was no significant correlation between any of the dispositional items and composite baseline wrongness in either society, with the exception of the Caused by the Situation question in the Temporal Distance context in Los Angeles (see Table 3), which appears to be driven by the Stranger Theft and Market Cheating scenarios (see Table S9; for correlations between causal appraisals and wrongness ratings within each context condition for each scenario, see SI Tables S7–S10.).

**Table 3:** Correlations between composite causal attributions and wrongness ratings.

Context	Situation as cause	*p*	Others would have	*p*
**Los Angeles (*N* = 69)**				
Baseline	0.23	0.055	− 0.02	0.862
Authority consent	0.13	0.306	0.15	0.211
Spatial distance	0.21	0.086	0.05	0.700
Temporal distance	0.25	0.040	0.12	0.327
**Seoul (*N* = 52)**				
Baseline	− 0.10	0.503	− 0.12	0.396
Authority consent	0.03	0.841	0.12	0.401
Spatial distance	0.04	0.782	0.04	0.802
Temporal distance	− 0.03	0.839	0.04	0.764

See SI for correlations within each context for each scenario.

### Null effect of society on reputational consequences of transgression

A between-subjects ANOVA revealed that, despite the large differences in dispositional versus situational causal attribution, there was no significant difference in ratings of the average reputational consequences of transgression between the Seoul sample (*M* = 2.59, *SD* = 0.83) and the Los Angeles sample (*M* = 2.43, *SD* = 0.77), *p* = 0.282. Follow-up tests likewise revealed no significant differences between societies in estimated reputational consequences in any save one of the individual scenarios, Domestic Battery, which the Los Angeles sample rated as having lower reputational consequences than did the South Korean sample (see SI Table S11).

#### Society, causal attribution and reputational consequences of transgression

We next assessed correlations between estimated reputational consequences and each measure of causal attribution. Neither of the composited causal attribution ratings was significantly associated with estimated reputational consequences when analyzed within each society separately (Others Would Also Transgress: *Seoul*: *r*(51) = 0.15, *p* = 0.292; *Los Angeles*: *r*(68) = 0.04, *p* = 0.749; Caused by the Situation: *Seoul*: *r*(51) = 0.25, *p* = 0.078; *Los Angeles*: *r*(68) = 0.22, *p* = 0.064. (However, see SI Table S12 for analyses revealing exceptions: two scenarios in which positive correlations were observed in the Los Angeles sample, and one scenario for which a positive association was observed in the Seoul sample).

## Discussion

We assessed the role of context on assessments of moral wrongness in two societies, as well as the potential relationship between cultural differences in perceptions of wrongness and in appraisals of the causes of transgressive acts. With respect to the effects of context, we replicated the moral parochialism effect obtained in prior cross-cultural studies conducted outside of East Asia. Here, in both Seoul and Los Angeles, transgressions presented as occurring at spatial or temporal distance, or with the consent of local authorities, were evaluated as less wrong relative to baseline. With respect to causal attribution, we replicated prior findings^20,21^ that East Asian individuals evaluate moral transgressions as caused by interactions between individuals and their situational circumstances moreso than do Westerners, who tend to attribute social behavior to the dispositions of the actors, independent of context. One of our convergent measures of causal appraisal asked whether “almost no one” or “almost anyone” would morally transgress under the circumstances in the scenario. Although participants in Seoul tended to indicate that most people would commit immoral acts to a strikingly greater extent than did Los Angeles participants, this need not be interpreted as a pessimistic view of human nature, but rather an appreciation that dispositions may exert relatively less causal force than the circumstances in which they are embedded.

There were no differences between the Seoul and Los Angeles samples in the estimated degree of harm to the transgressor’s reputation within their community as a moral person, despite sizable differences in attributing the harmful actions to the transgressor’s disposition versus contextual factors. A variety of theoretical perspectives argue that human attention to reputation evolved to enhance partner choice in cooperative ventures; to increase the costs of defection and non-adherence to group-functional norms; and to lower the costs of punishing non-cooperators^7,8^. In all of these approaches, the locus of information is the agent—reputations must adhere to individual actors as a function of their past behavior if others are to predict, preempt, or respond to their future behavior. Seen in this light, the similarity in reputation assessments across our Korean and U.S. participants may appear puzzling given the former’s greater assignation of causality to situational factors versus unique attributes of the agent. Conceivably, this lack of association between reputation and causal appraisals may be explained by the function of reputation as a predictor of an agent’s future behavior. Individuals who find themselves in situations that lead to norm transgressions are likely to encounter comparable situations in the future and presumably respond similarly, hence observers may gain utility from reputational information whether ascribing the individual’s behavior to disposition or circumstances.

The causal appraisal measures used in our present research assess dispositional versus situational attributions in a face-valid way, but do not capture potentially related societal differences in the amount of information considered when evaluating the causes of transgressions, or the complexity of relational representations. Employing methods designed to test societal differences in the amount of information considered, Choi and colleagues found that Koreans took more relational information into consideration when attempting to understand a murderer’s motive when compared to U.S. participants^21^. Following Choi and colleagues, future work incorporating more complex, information-rich transgression scenarios and measures of how participants interact with informational features would help to clarify the extent to which the present differences in causal appraisal (and possibly in the relative magnitudes of moral parochialism) observed between our Seoul and Los Angeles samples owe to a divergence in informationally rich cognitive processing.

The Seoul sample assessed the composite transgressions occurring at a spatial or temporal distance as less wrong relative to baseline than did the Los Angeles sample, in significant interactions between society and the context manipulation. These findings are consistent with the possibility that situational appraisals of the causes of transgressive harms potentiate parochial moral intuitions regarding the severity of their wrongness, as manipulating the context in which a transgression occurs inherently involves situational considerations. However, for two reasons, this interpretation should be treated cautiously. First, there was no interaction between society and situational context with regard to the consent of local authorities, yet one would expect authority consent to temper condemnation as a contextual factor if situational causal attribution moderates wrongness judgments, all else being equal. Second, a comparable pattern of heightened moral parochialism was previously observed with regard to the magnitude of the effect of spatial or temporal distance—but not authority consent—on wrongness ratings between a U.S. sample recruited in California cities (comparable to the present Los Angeles sample) and a Ukrainian sample recruited in the town of Storozhnitsa^10^. There are no findings, to our knowledge, indicating that individuals in Western Ukraine tend to appraise the causes of moral transgressions in a relatively situational rather than dispositional fashion, raising the likelihood that an as yet unspecified alternative factor shared by Korea and Ukraine may parsimoniously account for the somewhat greater effect of the spatial and temporal distance manipulations. Alternately, the slightly greater moral parochialism observed in the present Seoul sample may indeed owe to differences in causal appraisal, whereas the greater moral parochialism observed in Storozhnitsa may be driven by other factors. Given the uncertainties, and the novel nature of the finding, the modest effect of society on the magnitude of moral parochialism should be considered preliminary pending replication, and the possible role of causal attribution style to this difference should be clarified in future research.

The present research on appraisals of transgressive acts motivates future inquiry into moral parochialism in appraisals of the causes of morally praiseworthy acts. With respect to moral parochialism, the cost/benefit tradeoffs incentivizing attenuated condemnation of harmful acts may not apply equivalently to praise of benevolent acts. For example, the moral parochialism hypothesis entails that the overt consent of local authorities should heighten praise of helpful acts, but the potential rewards of praise in this circumstance would appear lower in magnitude relative to the potential costs of condemning an act of harm despite it being countenanced by local authorities. Signaling one’s moral approval of acts occurring at spatial remove may similarly generate less benefits than would condemnation of harmful acts, as the latter may be taken by in-group members as a cue of in-group chauvinism, whereas the former may be taken as a cue of investment in out-group communities at the expense of commitment to the in-group. Given such potential differences in the functional consequences of praise versus condemnation under contexts of authority consent or spatial distance, it is an empirical question whether judgments of the rightness of prosocial acts evince contextual contingency to a comparable extent to the wrongness judgments observed here and in the previous research on moral parochialism. Prior work indicates that East Asian societies similarly understand prosocial acts as rooted in situational determinants to a greater extent than Western individuals prone to the fundamental attribution error^17–21^. Future work modeled after the present design might explore whether and to what degree this difference in causal appraisal influences the effects of context on judgments of the rightness of praiseworthy deeds.

We have discussed the functional logic of moral parochialism in a manner which, at the proximate level of description, is compatible with a role for affective responses in contextual reductions in judgments of wrongness. However, although considerable prior work links moral judgment with emotion^24^, the observed effects of context on wrongness evaluations need not be driven by corresponding effects on state emotion, and moral cognition incorporates both affective and nonaffective pathways^25^. For example, some individuals may disregard their feelings of distress when judging the wrongness of a transgression upon learning that local authorities approve of this type of behavior, thereby rating the act as less wrong despite feeling it to be equivalently upsetting as at baseline. Alternatively, the consent of local authorities may potentiate a diminution of reflexive emotional reactions. Similarly, transgressions occurring in a remote land or the deep past may elicit attenuated affective responses relative to baseline, resulting in diminutions in wrongness ratings. With respect to societal differences in the role of affect, the more cognitively sophisticated, contextually situated mode of representing the causes of transgressions evident in East Asian societies may reduce the degree to which state affect is elicited and/or integrated into wrongness judgments. A complementary line of research indicates that East Asian societies value low-arousal emotional states over high-arousal states^26^, raising the possibility that a situational mode of reflective appraisal may relate to regulation of arousal in response to transgressive harms, the better to dispassionately appraise their causes. Future cross-cultural work incorporating affective measures is required to understand the role of affect in both universal and varying aspects of moral parochialism.

Following Fessler et al.^10^, we utilized prototypical transgression scenarios without leavening motives for the perpetrators (e.g., the man who commits domestic battery does so without provocation, to vent frustration regarding a misfortune he understands not to be the fault of his spouse). Prior research indicates that in contexts of more complex, ambiguous harm scenarios (e.g., harsh interrogation of suspected terrorists with the intent of saving innocent lives), manipulations of authority consent, spatial distance or temporal distance may prompt participants to infer mitigating contextual rationales^27,28^. When approaching such complex scenarios, participants appear to judge acts of harm more permissible insofar as a greater good is served, the infliction of harm is deemed just, or the recipient of the harm had provided informed consent beforehand, among other justifications^27^. For example, manipulating the consent of military authorities can lead participants to approve of the torture of suspected terrorists, and appears related to the utilitarian inference that inflicting suffering will effectively save lives^28^. This prior work raises the possibility that participants in the present studies evinced moral parochialism because they similarly inferred mitigating contextual factors when asked to evaluate acts of harm such as theft, cheating, defamation, or unprovoked battery when committed far away, long ago, or with the consent of local authorities. However, were this the case, then the strikingly large societal differences we observed in holistic, situational attributions of the causes of these transgressions should correlate with correspondingly striking societal differences in appraisals of their wrongness. To the contrary, we observed wrongness reduction in both societies (notwithstanding modest differences in the effects of spatial and temporal distance), and there was no consistent pattern of correlation between holistic causal appraisals and wrongness ratings in either society. Although in some scenarios, and in some contexts, it may be the case that participants reduced their wrongness ratings because they inferred mitigating factors, it does not appear plausible that this explanation could account for the near-uniform reductions observed across scenarios in both societies, particularly given the reliance on scenarios in which the salience of potential mitigating justifications was minimized. Assessments of the contribution of contextual factors thus appear largely orthogonal to appraisals of the wrongness of simple, prototypical acts of harm such as those utilized here. Future research might explore whether societal differences in causal appraisal drive differences in wrongness evaluations of more complex, morally ambiguous acts. Individuals may or may not possess introspective access to rationales supporting their wrongness judgments, and those rationales which can be articulated may be post hoc confabulations to justify moral intuitions^24^. Further research is required to illuminate the proximate mechanisms supporting parochial shifts in moral judgment.

The present findings with regard to both moral parochialism and East/West differences in appraisals of the causes of moral behavior conceptually replicate previous research, yet additional replication will be important given their theoretical and social ramifications. Although our use of an in-person interview format arguably provides greater ecological validity with regard to the interpersonal social contexts in which moral intuitions and pronouncements have traditionally occurred, this approach is time-intensive. Accordingly, these designs might be modified for online research. In addition to logistical advantages, an online paradigm would better reflect moral evaluations that occur during internet-mediated exposure to information about transgressive acts. Replication efforts might also add additional transgression scenarios to those used here, given the likelihood of cross-cultural differences in the meaning of these acts. Demonstrating that the moral parochialism and causal attribution effects observed in the present design are replicable when evaluating other transgressive acts would help to confirm that our findings are generalizable, and are not artifacts of culturally inequivalent understandings of certain scenarios. Finally, our two field samples were recruited on the streets of large urban centers, and may not be representative of the heterogenous societies in which they are embedded. Future work attempting to better approximate representative samples would be valuable.

## Conclusion

Previous work has documented moral parochialism—the reduction in appraisals of moral wrongness associated with spatial or temporal distance or the consent of authorities—in samples from several large-scale Western societies and a number of small-scale societies. Absent from this corpus, however, were members of any large-scale societies of East Asia, populations known to differ from those of the West along a variety of psychological dimensions. We both replicated prior results in the urban U.S. and found moral parochialism in urban South Korea; we also replicated prior work showing that members of East Asian societies predominantly attribute moral transgressions to situational factors, whereas members of Western societies tend to attribute transgressions to the intrinsic dispositions of transgressors. Importantly, the fact that we find roughly comparable levels of moral parochialism in the two societies studied despite substantive differences in the attribution of causal factors adds to the body of evidence supporting the theorized universality of moral parochialism across disparate psycho-cultural systems. If moral parochialism indeed reflects core design features of the species-typical evolved mechanisms underlying human moral reasoning, then this pattern should continue to obtain as investigators expand the number and variety of populations studied.

## Supplementary Information

Supplementary Information.

## Data Availability

The dataset and full materials are available on the Open Science Framework: https://osf.io/jq2vm/.
