# Supplementary material for: Moral parochialism and causal appraisal of transgressive harm in Seoul and Los Angeles
Source: Sci Rep. 2022 Aug 20;12:14227. doi: 10.1038/s41598-022-18521-0 (PMC9392729; doi:10.1038/s41598-022-18521-0)
Supplement: Supplementary file 1 — Supplementary Information. [file 41598_2022_18521_MOESM1_ESM.docx]

**Supplementary Information**

**to accompany**

**Moral Parochialism and Causal Appraisal of Transgressive Harm**

**in Seoul and Los Angeles**

Colin Holbrook, Leehyun Yoon, Daniel M.T. Fessler, Cody Moser, Shairy Jimenez Delgado, and Hackjin Kim

**Table of Contents**

**Effects of society and context on perceived wrongness** **of each transgression scenario**

1. Stranger Theft

2. Domestic Battery

3. Unintentional Harm

4. Market Cheating

5. Defamation

6. Injustice

**Study Materials**

14. Wrongness Measure

15. Causal Attribution Measures

16. Four Orders of Scenario and Context Question Presentation

17. Los Angeles Study Protocol, Order 1

38. Seoul Study Protocol, Order 1

**Tables:**

1. *Table S1.* Transgression Context and Wrongness Ratings by Society: Stranger Theft

2. *Table S2.* Transgression Context and Wrongness Ratings by Society: Domestic Battery

3. *Table S3.* Transgression Context and Wrongness Ratings by Society: Unintentional Harm

4. *Table S4.* Transgression Context and Wrongness Ratings by Society: Market Cheating

5. *Table S5.* Transgression Context and Wrongness Ratings by Society: Defamation

6. *Table S6.* Transgression Context and Wrongness Ratings by Society: Injustice

7. *Table S7.* Zero-order Correlations Between Causal Appraisals and Baseline Wrongness by Scenario and Society

8. *Table S8.* Zero-order Correlations Between Causal Appraisals and Wrongness at Spatial Distance by Scenario and Society

9. *Table S9.* Zero-order Correlations Between Causal Appraisals and Wrongness at Temporal Distance by Scenario and Society

10. *Table S10.* Zero-order Correlations Between Causal Appraisals and Wrongness with Authority Consent by Scenario and Society

11. *Table S11.* Reputational Consequences of Transgression by Scenario and by Society

12. *Table S12.* Zero-order Correlations Between Causal Appraisals and Reputational Consequences by Scenario and Society

13. *Table S13.* Demographic Information

The dataset, orders 2-4 of the study protocol, and the back-translation of the Korean protocol into English may be accessed at https://osf.io/jq2vm/.

**Stranger Theft**

We conducted a 2 (society) by 4 (transgression context) mixed-model ANOVA, with society as the between-subjects variable and context as the within-subjects variable. As hypothesized, there was a main effect of transgression context, *F*(3, 357) = 9.68, *p* < .001, *η_p_*^2^ = .08, with the composite transgressions rated significantly more wrong in the Baseline condition than in the Authority Consent (*p* < .001, *η_p_*^2^ = .14), Spatial Distance (*p* < .001, *η_p_*^2^ = .17), and Temporal Distance (*p* < .001, *η_p_*^2^ = .16) conditions, pooling both societies. This main effect was qualified by two significant interaction effects between society and context condition, wherein the Korean participants rated the Stranger Theft transgression as less wrong relative to baseline in the Spatial Distance context, *F*(1, 119) = 9.81, *p* = .002, *η_p_*^2^ = .08, and in the Temporal Distance context, *F*(1, 119) = 5.26, *p* = .024, *η_p_*^2^ = .04, than did American participants. There was no such interaction with regard to the context of Authority Consent, *p* = .894, nor were there main effects of society on ratings in any context, *p*s .194 - .438.

| Table S1 | |  |  |  | |  | |  | |  |  |
| --- | --- | --- | --- | --- | --- | --- | --- | --- | --- | --- | --- |
| Transgression Context and Wrongness Ratings by Society: Stranger Theft | | | | | | | |  |  |  |  |
| *Context* | | | *M* | | *SD* | *p* | *η_p_^2^* | |  |  |  |
| Los Angeles (*N* = 69) | Baseline | | 1.75 | | .93 |  |  | |  |  |  |
|  | Authority Consent | | 2.26 | | 1.50 | .003 | .13 | |  |  |  |
|  | Spatial Distance | | 1.91 | | 1.13 | .040 | .06 | |  |  |  |
|  | Temporal Distance | | 2.00 | | 1.27 | .043 | .06 | |  |  |  |
| Seoul (*N* = 52) | Baseline | | 1.52 | | 1.04 |  |  | |  |  |  |
|  | Authority Consent | | 2.06 | | 1.31 | .002 | .18 | |  |  |  |
|  | Spatial Distance | | 2.25 | | 1.52 | <.001 | .24 | |  |  |  |
|  | Temporal Distance | | 2.21 | | 1.40 | <.001 | .27 | |  |  |  |

*Note.* Lower ratings indicate appraisals of the transgressive act as more wrong.

**Domestic Battery**

We conducted a 2 (society) by 4 (transgression context) mixed-model ANOVA, with society as the between-subjects variable and context as the within-subjects variable. As hypothesized, there was a main effect of transgression context, *F*(3, 357) = 14.94, *p* < .001, *η_p_*^2^ = .11, with the composite transgressions rated significantly more wrong in the Baseline condition than in the Authority Consent (*p* = .002, *η_p_*^2^ = .08), Spatial Distance (*p* < .001, *η_p_*^2^ = .16), and Temporal Distance (*p* < .001, *η_p_*^2^ = .22) conditions, pooling both societies. This main effect was qualified by a significant interaction between society and context condition, wherein the Korean participants rated the Domestic Battery transgression as less wrong relative to baseline than American participants in the Temporal Distance context, *F*(1, 119) = 5.87, *p* = .017, *η_p_*^2^ = .05. There was no such interaction with regard to the context of Authority Consent, *p* = .505, or Spatial Distance, *p* = .147. There was also a main effect of society such that the Korean sample rated the transgression as less wrong on average in the context of Spatial Distance, *F*(1, 119) = 5.95, *p* = .016, *η_p_*^2^ = .05, 95% CI [.09, .89], and in the context of Temporal Distance, *F*(1, 119) = 9.06, *p* = .003, *η_p_*^2^ = .07, 95% CI [.27, 1.30], relative to the American sample (see Table S2). There were no such effects of society on baseline wrongness ratings, *p* = .095, or wrongness within the context of Authority Consent, *p* = .121.

| Table S2 | |  |  |  | |  | |  | | |  |  |
| --- | --- | --- | --- | --- | --- | --- | --- | --- | --- | --- | --- | --- |
| Transgression Context and Wrongness Ratings by Society: Domestic Battery | | | | | | | | |  |  |  |  |
| *Context* | | | *M* | | *SD* | *p* | *η_p_^2^* | | |  |  |  |
| Los Angeles (*N* = 69) | Baseline | | 1.32 | | .58 |  |  | | |  |  |  |
|  | Authority Consent | | 1.59 | | 1.20 | .053 | .05 | | |  |  |  |
|  | Spatial Distance | | 1.62 | | .97 | .005 | .11 | | |  |  |  |
|  | Temporal Distance | | 1.73 | | 1.14 | .001 | .15 | | |  |  |  |
| Seoul (*N* = 52) | Baseline | | 1.52 | | .73 |  |  | | |  |  |  |
|  | Authority Consent | | 1.94 | | 1.22 | .019 | .10 | | |  |  |  |
|  | Spatial Distance | | 2.10 | | 1.16 | .001 | .19 | | |  |  |  |
|  | Temporal Distance | | 2.51 | | 1.73 | <.001 | .27 | | |  |  |  |

*Note.* Lower ratings indicate appraisals of the transgressive act as more wrong.

**Unintentional Harm**

We conducted a 2 (society) by 4 (transgression context) mixed-model ANOVA, with society as the between-subjects variable and context as the within-subjects variable. As hypothesized, there was a main effect of transgression context, *F*(3, 357) = 9.07, *p* < .001, *η_p_*^2^ = .07, with the composite transgressions rated significantly more wrong in the Baseline condition than in the Authority Consent (*p* = .001, *η_p_*^2^ = .10), Spatial Distance (*p* < .001, *η_p_*^2^ = .17), and Temporal Distance (*p* < .001, *η_p_*^2^ = .14) conditions, pooling both societies. There were no significant interactions between society and context condition, *p*s .447 - .853. There were also main effects of society such that the Korean sample rated the transgression as less wrong on average at Baseline, *F*(1, 119) = 9.92, *p* = .002, *η_p_*^2^ = .08, 95% CI [.25, 1.10], in the context of Spatial Distance, *F*(1, 119) = 10.43, *p* = .002, *η_p_*^2^ = .08, 95% CI [.31, 1.27], and in the context of Temporal Distance, *F*(1, 119) = 5.11, *p* = .026, *η_p_*^2^ = .04, 95% CI [.08, 1.19], relative to the American sample (see Table S3). There were no such effects of society on perceived wrongness within the context of Authority Consent, *p* = .115.

| Table S3  Transgression Context and Wrongness Ratings by Society: Unintentional Harm | | | | | |  |
| --- | --- | --- | --- | --- | --- | --- |
| *Context* | | *M* | *SD* | *p* | *η_p_^2^* | |
| Los Angeles (*N* = 69) | Baseline | 2.77 | 1.15 |  |  | |
|  | Authority Consent | 3.32 | 1.54 | <.001 | .21 | |
|  | Spatial Distance | 3.18 | 1.46 | <.001 | .18 | |
|  | Temporal Distance | 3.26 | 1.55 | <.001 | .21 | |
| Seoul (*N* = 52) | Baseline | 3.44 | 1.18 |  |  | |
|  | Authority Consent | 3.80 | 1.78 | .140 | .04 | |
|  | Spatial Distance | 3.96 | 1.14 | .003 | .16 | |
|  | Temporal Distance | 3.89 | 1.49 | .029 | .09 | |

*Note.* Lower ratings indicate appraisals of the transgressive act as more wrong.

**Market Cheating**

We conducted a 2 (society) by 4 (transgression context) mixed-model ANOVA, with society as the between-subjects variable and context as the within-subjects variable. As hypothesized, there was a main effect of transgression context, *F*(3, 357) = 11.92, *p* < .001, *η_p_*^2^ = .09, with the composite transgressions rated significantly more wrong in the Baseline condition than in the Authority Consent (*p* < .001, *η_p_*^2^ = .14), Spatial Distance (*p* < .001, *η_p_*^2^ = .15), and Temporal Distance (*p* < .001, *η_p_*^2^ = .19) conditions, pooling both societies. There were no significant interactions between society and context condition, *p*s .182 - .664. There were no significant main effects of society on wrongness ratings within the contexts of Authority Consent, *p* = .345, Spatial Distance, *p* = .831, or Temporal Distance, *p* = .501, although there was a nonsignificant trend in which Koreans rated the Market Cheating transgression as more wrong at Baseline than did U.S. participants, *F*(1, 119) = 3.70, *p* = .057, *η_p_*^2^ = .03, 95% CI [-.74, .01].

| Table S4  Transgression Context and Wrongness Ratings by Society: Market Cheating | | | | | |  |
| --- | --- | --- | --- | --- | --- | --- |
| *Context* | | *M* | *SD* | *p* | *η_p_^2^* | |
| Los Angeles (*N* = 69) | Baseline | 2.03 | 1.08 |  |  | |
|  | Authority Consent | 2.49 | 1.45 | .003 | .12 | |
|  | Spatial Distance | 2.41 | 1.35 | .009 | .10 | |
|  | Temporal Distance | 2.51 | 1.36 | <.001 | .21 | |
| Seoul (*N* = 52) | Baseline | 1.66 | .96 |  |  | |
|  | Authority Consent | 2.23 | 1.58 | .004 | .15 | |
|  | Spatial Distance | 2.34 | 1.71 | .001 | .21 | |
|  | Temporal Distance | 2.32 | 1.74 | .002 | .17 | |

*Note.* Lower ratings indicate appraisals of the transgressive act as more wrong.

**Defamation**

We conducted a 2 (society) by 4 (transgression context) mixed-model ANOVA, with society as the between-subjects variable and context as the within-subjects variable. As hypothesized, there was a main effect of transgression context, *F*(3, 357) = 5.78, *p* = .001, *η_p_*^2^ = .05, with the composite transgressions rated significantly more wrong in the Baseline condition than in the Authority Consent (*p* = .001, *η_p_*^2^ = .09), Spatial Distance (*p* = .003, *η_p_*^2^ = .07), and Temporal Distance (*p* = .005, *η_p_*^2^ = .06) conditions, pooling both societies. This main effect was qualified by a significant interaction between society and context condition, wherein the Korean participants rated the Domestic Battery transgression as less wrong relative to baseline than American participants in the Spatial Distance context, *F*(1, 119) = 9.17, *p* = .003, *η_p_*^2^ = .07, and in the Temporal Distance context, *F*(1, 119) = 4.23, *p* = .042, *η_p_*^2^ = .03. There was no such interaction with regard to the context of Authority Consent, *p* = .601. There were no significant main effects of society on wrongness ratings at Baseline or within the contexts of Authority Consent or Temporal Distance, *p*s .270 - .558, although there was a nonsignificant trend in which Koreans rated the Domestic Battery transgression as less wrong in the Spatial Distance context than did U.S. participants, *F*(1, 119) = 3.54, *p* = .062, *η_p_*^2^ = .01, 95% CI [-.02, .91].

| Table S5  Transgression Context and Wrongness Ratings by Society: Defamation | | | | | |  |
| --- | --- | --- | --- | --- | --- | --- |
| *Context* | | *M* | *SD* | *p* | *η_p_^2^* | |
| Los Angeles (*N* = 69) | Baseline | 2.17 | .97 |  |  | |
|  | Authority Consent | 2.61 | 1.44 | .005 | .11 | |
|  | Spatial Distance | 2.17 | 1.18 | 1.000 | .00 | |
|  | Temporal Distance | 2.25 | 1.33 | .533 | .01 | |
| Seoul (*N* = 52) | Baseline | 2.07 | 1.01 |  |  | |
|  | Authority Consent | 2.39 | 1.36 | .060 | .07 | |
|  | Spatial Distance | 2.62 | 1.40 | .002 | .17 | |
|  | Temporal Distance | 2.52 | 1.35 | .003 | .16 | |

*Note.* Lower ratings indicate appraisals of the transgressive act as more wrong.

**Injustice**

We conducted a 2 (society) by 4 (transgression context) mixed-model ANOVA, with society as the between-subjects variable and context as the within-subjects variable. As hypothesized, there was a main effect of transgression context, *F*(3, 357) = 6.08, *p* < .001, *η_p_*^2^ = .05, with the composite transgressions rated significantly more wrong in the Baseline condition than in the Authority Consent (*p* = .038, *η_p_*^2^ = .04), Spatial Distance (*p* = .001, *η_p_*^2^ = .09), and Temporal Distance (*p* < .001, *η_p_*^2^ = .11) conditions, pooling both societies. This main effect was qualified by a significant interaction between society and context condition, wherein the Korean participants did not rate the transgression as less wrong relative to baseline than American participants in the Authority Consent context, *F*(1, 119) = 4.03, *p* = .047, *η_p_*^2^ = .03. There was no such interaction with regard to the contexts of Spatial Distance, *p* = .263, or Temporal Distance, *p* = .216. There were no significant main effects of society on wrongness ratings at Baseline or within the contexts of Spatial or Temporal Distance, *p*s .270 - .983, although there was a nonsignificant trend in which Koreans rated the Injustice transgression as more wrong in the Authority Consent context than did U.S. participants, *F*(1, 119) = 3.83, *p* = .053, *η_p_*^2^ = .03, 95% CI [-.92, .01].

| Table S6  Transgression Context and Wrongness Ratings by Society: Injustice | | | | | |  |
| --- | --- | --- | --- | --- | --- | --- |
| *Context* | | *M* | *SD* | *p* | *η_p_^2^* | |
| Los Angeles (*N* = 69) | Baseline | 1.84 | .92 |  |  | |
|  | Authority Consent | 2.30 | 1.42 | .002 | .13 | |
|  | Spatial Distance | 2.10 | 1.26 | .038 | .06 | |
|  | Temporal Distance | 2.12 | 1.10 | .036 | .08 | |
| Seoul (*N* = 52) | Baseline | 1.84 | 1.15 |  |  | |
|  | Authority Consent | 1.85 | 1.06 | .956 | .00 | |
|  | Spatial Distance | 2.37 | 1.55 | .020 | .10 | |
|  | Temporal Distance | 2.39 | 1.57 | .010 | .12 | |

*Note.* Lower ratings indicate appraisals of the transgressive act as more wrong.

| Table S7 |  |  |  |  |  |  |  |
| --- | --- | --- | --- | --- | --- | --- | --- |

Zero-order Correlations Between Causal Appraisals and Baseline Wrongness by Scenario and Society

| *Scenario* | *Caused by Situation*  Seoul  *r*, *p* | *Caused by Situation*  Los Angeles  *r*, *p* | *Others Would Also*  Seoul  *r*, *p* | *Others Would Also*  Los Angeles  *r*, *p* |
| --- | --- | --- | --- | --- |
| *Stranger Theft* | -.03, .842 | .31, .010* | -.05, .731 | .14, .256 |
| *Domestic Battery* | .13, .369 | -.12, .338 | .10, .487 | -.16, .187 |
| *Unintentional Harm* | .10, .491 | .35, .003* | .08, .582 | .20, .101 |
| *Market Cheating* | .07, .637 | .16, .182 | .24, .088 | .07, .544 |
| *Defamation* | .10, .488 | -.04, .769 | -.02, .864 | .00, .998 |
| *Injustice* | .20, .102 | .20, .102 | .01, .912 | .01, .912 |

*Note.* Seoul *N* = 52; Los Angeles *N* = 69; * *p* < .05.

| Table S8 |  |  |  |  |  |  |  |
| --- | --- | --- | --- | --- | --- | --- | --- |

Zero-order Correlations Between Causal Appraisals and Wrongness at Spatial Distance by Scenario and Society

| *Scenario* | *Caused by Situation*  Seoul  *r*, *p* | *Caused by Situation*  Los Angeles  *r*, *p* | *Others Would Also*  Seoul  *r*, *p* | *Others Would Also*  Los Angeles  *r*, *p* |
| --- | --- | --- | --- | --- |
| *Stranger Theft* | -.04, .778 | .39, .001* | -.15, .287 | .20, .093 |
| *Domestic Battery* | .31, .027* | -.07, .565 | .17, .237 | -.19, .123 |
| *Unintentional Harm* | .19, .186 | .24, .049* | .07, .631 | .17, .157 |
| *Market Cheating* | .18, .197 | .30, .012* | .19, .174 | .09, .451 |
| *Defamation* | -.02, .893 | -.03, .808 | .04, .805 | .19, .111 |
| *Injustice* | -.17, .228 | .12, .344 | -.26, .062 | -.06, .611 |

*Note.* Seoul *N* = 52; Los Angeles *N* = 69; * *p* < .05.

| Table S9 |  |  |  |  |  |  |  |
| --- | --- | --- | --- | --- | --- | --- | --- |

Zero-order Correlations Between Causal Appraisals and Wrongness at Temporal Distance by Scenario and Society

| *Scenario* | *Caused by Situation*  Seoul  *r*, *p* | *Caused by Situation*  Los Angeles  *r*, *p* | *Others Would Also*  Seoul  *r*, *p* | *Others Would Also*  Los Angeles  *r*, *p* |
| --- | --- | --- | --- | --- |
| *Stranger Theft* | -.04, .788 | .40, .001* | -.23, .102 | .22, .076 |
| *Domestic Battery* | .17, .222 | -.06, .610 | .02, .884 | -.21, .092 |
| *Unintentional Harm* | .05, .752 | .19, .114 | .02, .907 | .13, .272 |
| *Market Cheating* | .05, .731 | .35, .003* | .04, .769 | .32, .007* |
| *Defamation* | .00, .998 | -.01, .959 | -.03, .824 | .16, .198 |
| *Injustice* | -.17, .222 | .12, .338 | -.21, .137 | .07, .590 |

*Note.* Seoul *N* = 52; Los Angeles *N* = 69; * *p* < .05.

| Table S10 |  |  |  |  |  |  |  |
| --- | --- | --- | --- | --- | --- | --- | --- |

Zero-order Correlations Between Causal Appraisals and Wrongness with Authority Consent by Scenario and Society

| *Scenario* | *Caused by Situation*  Seoul  *r*, *p* | *Caused by Situation*  Los Angeles  *r*, *p* | *Others Would Also*  Seoul  *r*, *p* | *Others Would Also*  Los Angeles  *r*, *p* |
| --- | --- | --- | --- | --- |
| *Stranger Theft* | -.17, .232 | .25, .038* | -.11, .453 | .15, .222 |
| *Domestic Battery* | .16, .273 | .08, .516 | .19, .185 | .01, 971 |
| *Unintentional Harm* | .09, .545 | .28, .019* | .09, .544 | .17 .162 |
| *Market Cheating* | .07, .641 | .18, .148 | .17, .236 | .19, .110 |
| *Defamation* | .09, .545 | -.17, .175 | -.04, .799 | .10, .394 |
| *Injustice* | .00, .978 | -.06, .626 | -.11, .432 | .11, .365 |

*Note.* Seoul *N* = 52; Los Angeles *N* = 69; * *p* < .05.

| Table S11 |  |  |  |  |  |  |  |
| --- | --- | --- | --- | --- | --- | --- | --- |

Reputational Consequences of Transgression by Scenario and by Society

| *Scenario* | Seoul  *N* = 52  *M* (*SD*) | | Los Angeles  *N* = 69  *M* (*SD*) | | *F* | *p* | | *η_p_^2^* | | *95% CIs* |
| --- | --- | --- | --- | --- | --- | --- | --- | --- | --- | --- |
| *Stranger Theft* | 2.20 (1.32) | | 2.19 (1.20) | | .00 | | .953 | | .00 | -.44, .47 |
| *Domestic Battery* | | 2.00 (1.26) | | 1.59 (.83) | 4.54 | .035 | | .04 | | .03, .78 |
| *Unintentional Harm* | | 3.90 (1.30) | | 3.54 (1.41) | 2.16 | .145 | | .02 | | -.13, .86 |
| *Market Cheating* | | 2.55 (1.64) | | 2.68 (1.22) | .26 | .610 | | .00 | | -.65, .38 |
| *Defamation* | | 2.42 (1.38) | | 2.49 (1.18) | .09 | .766 | | .00 | | -.53, .39 |
| *Injustice* | | 2.43 (1.42) | | 2.07 (1.14) | 2.40 | .124 | | .02 | | -.10, .82 |

*Note.* Lower ratings indicate appraisals of the transgressor’s reputation as that of a morally bad person; higher ratings indicate appraisals of the transgressor’s reputation reputation as that of a morally good person. *P* values, effect sizes, and 95% CIs reflect contrasts between the two societies.

| Table S12 |  |  |  |  |  |  |  |
| --- | --- | --- | --- | --- | --- | --- | --- |

Zero-order Correlations Between Causal Appraisals and Reputational Consequences by Scenario and Society

| *Scenario* | *Caused by Situation*  Seoul  *r*, *p* | *Caused by Situation*  Los Angeles  *r*, *p* | *Others Would Also*  Seoul  *r*, *p* | *Others Would Also*  Los Angeles  *r*, *p* |
| --- | --- | --- | --- | --- |
| *Stranger Theft* | .14, .325 | .45, <.001* | -.10, .505 | .28, .004* |
| *Domestic Battery* | .17, .227 | .06, .652 | .01, .960 | -.17, .174 |
| *Unintentional Harm* | .10, .475 | .35, .004* | .15, .283 | .24, .045* |
| *Market Cheating* | .16, .260 | .23, .059 | .36, .009* | .21, .081 |
| *Defamation* | .22, .117 | .20, .106 | -.10, .470 | .06, .599 |
| *Injustice* | -.02, .908 | -.15, .228 | .07, .613 | -.01, .945 |

*Note.* Seoul *N* = 52; Los Angeles *N* = 69; * *p* < .05.

| Table S13  Demographic Information | | | |  |
| --- | --- | --- | --- | --- |
|  |  | *Seoul* | *Los Angeles* | |
| Age (years) | Mean(SD) | 34.13(10.88) | 31.41(12.24) | |
| Political orientation | Mean(SD) | 2.82(.95) | 2.61(1.11) | |
| Sex | % Female | 42.3 | 52.2 | |
| Citizenship | % Korea | 96.2 | - | |
|  | % United States | - | 87.0 | |
|  | % Other | 3.8 | 8.7 | |
|  | % Not disclosed | - | 4.3 | |
| Country of birth | % Korea | 98.1 | 1.4 | |
|  | % United States | 1.9 | 87.0 | |
|  | % Other | - | 11.6 | |
| Ethnicity | % Korean | 100.0 | - | |
|  | % Asian (in U.S.) | - | 13.0 | |
|  | % White | - | 42.0 | |
|  | % Hispanic/Latino | - | 18.8 | |
|  | % Black | - | 10.1 | |
|  | % More than one | - | 11.6 | |
|  | % Other | - | 4.3 | |
| Education | % High school degree | 30.4 | 7.4 | |
|  | % Some college | - | 50.0 | |
|  | % Bachelor’s degree | 60.9 | 27.8 | |
|  | % Some graduate school | 4.3 | 7.4 | |
|  | % Advanced degree | 4.3 | 7.4 | |

*Note.* Political orientation was assessed using a 5-point scale (1 = *Very liberal*; 2 = *Slightly liberal*; 3 = *Moderate*; 4 = *Slightly conservative*; 5 = *Very conservative*).

**Wrongness Measure**

**(In the field studies, this scale was formatted to fill a landscape-oriented page using a 14-pt font for the text, printed and laminated for use in the field.)**


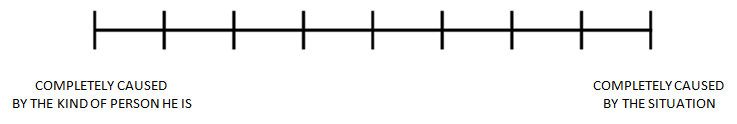


완전히 나쁘다 나쁘지도 좋지도 않다 완전히 좋다


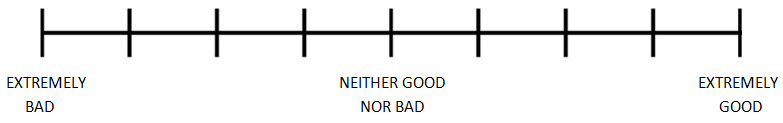


**Causal Attribution Measures**

**(In the field studies, these scales were formatted to fill landscape-oriented pages, using a 14-pt font for the text, printed on separate sheets. and laminated for use in the field.)**

*“*Now, how much do you feel that the [TRANSGRESSION OCCURRED] because of the kind of person that Richard is, in comparison to all of the other causes?


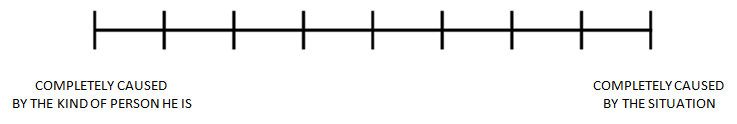


순전히

상황적인 요인들 때문에

일어난 일이다

순전히

그 사람이 어떤 종류의 사람이기 때문에

일어난 일이다


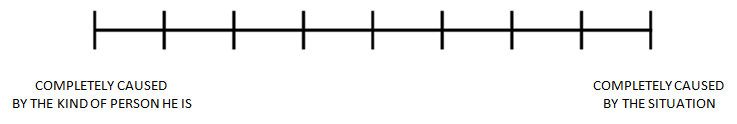


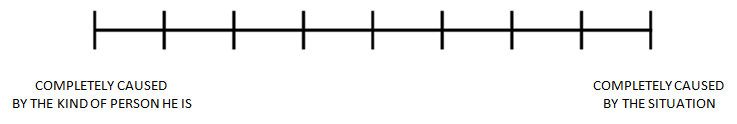


“Now, how much do you feel that other people would have been likely to [COMMIT RELEVANT TRANSGRESSION] if they found themselves in the exact same situation?”


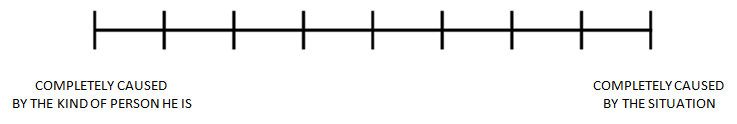


누구라도

그럴 것이다.

어느 누구도

그러지 않을 것이다.


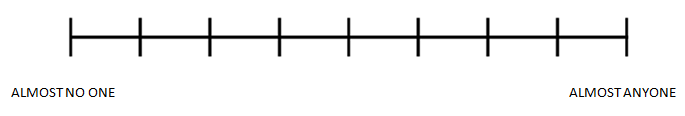


**Four Orders of Scenario and Context Question Presentation**

**Order 1:** Stealing from a Stranger [**MP:** A / T / S] [**CA:** Situ / O]; Battery [**MP:** T / S / A] [**CA:** O / Situ]; Unintentional Harm [**MP:** A / T / S] [**CA:** Situ / O]; Market Cheating [**MP:** T / S / A] [**CA:** O / Situ]; Defamation [**MP:** A / T / S] [**CA:** Situ / O]; Injustice [**MP:** T / S / A] [**CA:** O / Situ]

**Order 2:** Stealing from a Stranger [**MP:** T / S / A] [**CA:** O / Situ]; Battery [**MP:** A / T / S] [**CA:** Situ / O]; Unintentional Harm [**MP:** T / S / A] [**CA:** O / Situ]; Market Cheating [**MP:** A / T / S] [**CA:** Situ / O]; Defamation [**MP:** T / S / A] [**CA:** O / Situ]; Injustice [**MP:** A / T / S] [**CA:** Situ / O]

**Order 3:** Injustice [**MP:** A / T / S] [**CA:** Situ / O]; Defamation [**MP:** T / S / A] [**CA:** O / Situ]; Market Cheating [**MP:** A / T / S] [**CA:** Situ / O]; Unintentional Harm [**MP:** T / S / A] [**CA:** O / Situ]; Battery [**MP:** A / T / S] [**CA:** Situ / O]; Stealing from a Stranger [**MP:** T / S / A] [**CA:** O / Situ]

**Order 4:** Injustice [**MP:** T / S / A] [**CA:** O / Situ]; Defamation [**MP:** A / T / S] [**CA:** Situ / O]; Market Cheating [**MP:** T / S / A] [**CA:** O / Situ]; Unintentional Harm [**MP:** A / T / S] [**CA:** Situ / O]; Battery [**MP:** T / S / A] [**CA:** O / Situ]; Stealing from a Stranger [**MP:** A / T / S] [**CA:** Situ / O]

**MP =** Moral Parochialism

**CA =** Causal Attribution

**Moral Parochialism Items:**

A = Authority Dependence question

T = Temporal Distance question

S = Spatial Distance question

**Causal Attribution Items:**

O = Others would also Transgress question

Situ = Caused by the Situation question

# Los Angeles STUDY PROTOCOL, ORDER VERSION 1

# [Study – SCRIPT 1]

[Note to experimenter: The material in brackets is for your information and should not be read to the subjects]

**Instructions for Comprehension Checks for each Scenario**

[RA: If the participant gets one or more of the probes wrong (R1), say "Perhaps I did not make myself clear. Please let me read the story to you again;” then return to the story and re-read it once more. Then, go through the probes that the subject got wrong again, recording the responses in the R2 column (1 for a correct answer, 0 for an incorrect answer). After round 2, regardless of whether they still make mistakes, proceed to questions below.]

**[Preamble to Participants]**

I would like to read you some stories about events that occurred in an American community like this one, and then ask you some questions about each story. It will take about 30 minutes, and you will receive $10 as soon as we are finished. Participation is totally voluntary. If we start and then you decide that you would prefer not to participate, that is fine, and we can stop whenever you want. Are you willing to participate?

[If the participant agrees, go on:]

Before we begin, I would like for you to keep in mind that you will be asked similar questions about the different situations. Although these questions might begin to seem repetitive, please try to consider each one independently, as if it were the only question that you were asked.

# [#1: STEALING FROM A STRANGER]

James is a man from another American community. While camping near the town, James encounters a stranger from Tuva, a country that is very far away from here. The stranger does not speak English. After the stranger passes James, the stranger puts his sack down and walks down a small hill to wash in a stream. When the stranger is out of sight, James opens his sack and looks at the contents. He finds $700, takes the money and walks away quickly. The stranger does not realize his money has been taken until he is back home in his country, and he is then too far away to do anything about it.

# [#1: STEALING FROM A STRANGER: Comprehension Questions]

[RA: Read the following to the subject]

“Because it is important that I make myself clear, I’d like to make sure that you understood me. Could you please tell me what happened in the story I just told you?”

|  | **Concept** | **Mentioned in P’s 1^st^ summary? P1** | **Question if Not mentioned** | **Correct? R1** | **Correct? R2** |
| --- | --- | --- | --- | --- | --- |
| SC1 | Stole from stranger |  | From whom did James steal? |  |  |
| SC2 | Stranger different group |  | Was the stranger an American? |  |  |
| SC3 | Time delay in detection |  | Did the stranger detect that his money had been stolen while he was in the United States? |  |  |

# [#1: STEALING FROM A STRANGER: Questions]

| [Severity] | In your personal view, how good or bad is what James did? Please show me on this line. [Show subjects standard severity judgment scale. Be sure to record number from scale below.]  [Response:] _________ [record number from scale; If the participant indicates an intermediate point on the scale, record the nearest whole number value.] |
| --- | --- |

| [Justification] | Why? [Record notes on participant’s response here:] |
| --- | --- |

| [Reputation] | What would people in his community think of James? Would they think he is a good person or a bad person? Please show me on this line. [Show subjects standard severity judgment scale. Be sure to record number from scale below.]  [Response:] _________ [record number from scale; If the participant indicates an intermediate point on the scale, record the nearest whole number value.] |
| --- | --- |

| [Authority Dependence] | Suppose that the community leaders said that it is not bad to take things from strangers who do not live nearby and do not speak English. In your personal view, how good or bad would it be to do what James did, if the community leaders said that it was not bad? Please show me on this line. [Show subjects standard severity judgment scale. Be sure to record number from scale below.]  [Response:] _________ [record number from scale; If the participant indicates an intermediate point on the scale, record the nearest whole number value.] |
| --- | --- |

| [Long Ago] | What if this happened a long, long time ago, before your grandparents were born, even before their grandparents were born. In your personal view, how good or bad would it be to do what James did a very long time ago? Please show me on this line. [Show subjects standard severity judgment scale. Be sure to record number from scale below.]  [Response:] _________ [record number from scale; If the participant indicates an intermediate point on the scale, record the nearest whole number value.] |
| --- | --- |

| [Far Away] | What if this happened in a place very far from here, a place that no one in this town has ever visited, and I have never visited either. In your personal view, how good or bad would it be to do what James did if it happened very far from here? Please show me on this line. [Show subjects standard severity judgment scale. Be sure to record number from scale below.]  [Response:] _________ [record number from scale; If the participant indicates an intermediate point on the scale, record the nearest whole number value.] |
| --- | --- |

# [#2: BATTERY]

In this other American community, Stephen returns home feeling very angry because his belongings have been damaged in a storm. His wife, who he knows was not responsible for the damage to his belongings, greets him warmly as he enters the house. Because he is angry, he slaps her face very hard, causing her nose to bleed.

# [#2: BATTERY: Comprehension Questions]

[RA: Read the following to the subject]

“Because it is important that I make myself clear, I’d like to make sure that you understood me. Could you please tell me what happened in the story I just told you?”

|  | **Concept** | **Mentioned in P’s 1^st^ Summary? P1** | **Question if Not mentioned** | **Correct? R1** | **Correct? R2** |
| --- | --- | --- | --- | --- | --- |
| BC1 | Belongings damaged |  | What happened to Stephen’s belongings? |  |  |
| BC2 | Wife not responsible |  | Was Stephen’s wife responsible for the damage to A’s belongings? |  |  |
| BC3 | Slapped his wife |  | What did Stephen do to his wife? |  |  |

[RA: If the participant gets one or more of the probes wrong (R1), say "Perhaps I did not make myself clear. Please let me read the story to you again;” then return to the story and re-read it once more. Then, go through the probes that the subject got wrong again, recording the responses in the R2 column (1 for a correct answer, 0 for an incorrect answer). After round 2, regardless of whether they still make mistakes, proceed to questions below.]

# [#2: BATTERY: Questions]

| [Severity] | In your personal view, how good or bad is what Stephen did? Please show me on this line. [Show subjects standard severity judgment scale. Be sure to record number from scale below.]  [Response:] _________ [record number from scale; If the participant indicates an intermediate point on the scale, record the nearest whole number value.] |
| --- | --- |

| [Justification] | Why? [Record notes on participant’s response here:] |
| --- | --- |

| [Reputation] | What would people in his community think of Stephen? Would they think he is a good person or a bad person? Please show me on this line. [Show subjects standard severity judgment scale. Be sure to record number from scale below.]  [Response:] _________ [record number from scale; If the participant indicates an intermediate point on the scale, record the nearest whole number value.] |
| --- | --- |

| [Long Ago] | What if this happened a long, long time ago, before your grandparents were born, even before their grandparents were born. In your personal view, how good or bad would it be to do what Stephen did a very long time ago? Please show me on this line. [Show subjects standard severity judgment scale. Be sure to record number from scale below.]  [Response:] _________ [record number from scale; If the participant indicates an intermediate point on the scale, record the nearest whole number value.] |
| --- | --- |

| [Far Away] | What if this happened in place very far from here, a place that no one in this town has ever visited, and I have never visited either. In your personal view, how good or bad would it be to do what Stephen did if it happened very far from here? Please show me on this line. [Show subjects standard severity judgment scale. Be sure to record number from scale below.]  [Response:] _________ [record number from scale; If the participant indicates an intermediate point on the scale, record the nearest whole number value.] |
| --- | --- |

| [Authority Dependence] | Suppose that the community leaders said that it is not bad for a man to slap his wife if he is angry. In your personal view, how good or bad would it be to do what Stephen did, if the community leaders said that it was not bad? Please show me on this line.  [Show subjects standard severity judgment scale. Be sure to record number from scale below.]  [Response:] _________ [record number from scale; If the participant indicates an intermediate point on the scale, record the nearest whole number value.] |
| --- | --- |

# [#3: UNINTENTIONAL HARM]

In this other American community, Roger and Dan are walking on a very muddy path. Roger slips in the mud and accidentally knocks Dan down as he tries to regain his balance. Dan injures his arm in the fall. Dan knows that it was an accident. When he gets up Dan is very angry and hits Roger in the face.

# [#3: UNINTENTIONAL HARM: Comprehension Questions]

[RA: Read the following to the subject]

“Because it is important that I make myself clear, I’d like to make sure that you understood me. Could you please tell me what happened in the story I just told you?”

|  | **Concept** | **Mentioned in P’s 1^st^ Summary? P1** | **Question if Not mentioned** | **Correct? R1** | **Correct? R2** |
| --- | --- | --- | --- | --- | --- |
| HC1 | Intention |  | Did Roger knock Dan down on purpose? |  |  |
| HC2 | Injury |  | Was Dan injured in the fall? |  |  |
| HC3 | Physical Retribution |  | What did Dan do to Roger after he got back up? |  |  |

[RA: If the participant gets one or more of the probes wrong (R1), say "Perhaps I did not make myself clear. Please let me read the story to you again;” then return to the story and re-read it once more. Then, go through the probes that the subject got wrong again, recording the responses in the R2 column (1 for a correct answer, 0 for an incorrect answer). After round 2, regardless of whether they still make mistakes, proceed to questions below.]

# [#3: UNINTENTIONAL HARM: Questions]

| [Severity] | In your personal view, how good or bad is what Dan did? Please show me on this line. [Show subjects standard severity judgment scale. Be sure to record number from scale below.]  [Response:] _________ [record number from scale; If the participant indicates an intermediate point on the scale, record the nearest whole number value.] |
| --- | --- |

| [Justification] | Why? [Record notes on participant’s response here:] |
| --- | --- |

| [Reputation] | What would people in his community think of Dan? Would they think he is a good person or a bad person? Please show me on this line. [Show subjects standard severity judgment scale. Be sure to record number from scale below.]  [Response:] _________ [record number from scale; If the participant indicates an intermediate point on the scale, record the nearest whole number value.] |
| --- | --- |

| [Authority Dependence] | Suppose that the community leaders said that it is not bad for a man to hit another person if that person causes an injury, even when the injury was not caused intentionally. In your personal view, how good or bad would it be to do what Dan did, if the community leaders said that it was not bad? Please show me on this line.  [Show subjects standard severity judgment scale. Be sure to record number from scale below.]  [Response:] _________ [record number from scale; If the participant indicates an intermediate point on the scale, record the nearest whole number value.] |
| --- | --- |

| [Long Ago] | What if this happened a long, long time ago, before your grandparents were born, even before their grandparents were born. In your personal view, how good or bad would it be to do what Dan did a very long time ago? Please show me on this line. [Show subjects standard severity judgment scale. Be sure to record number from scale below.]  [Response:] _________ [record number from scale; If the participant indicates an intermediate point on the scale, record the nearest whole number value.] |
| --- | --- |

| [Far Away] | What if this happened in place very far from here, a place that no one in this town has ever visited, and I have never visited either. In your personal view, how good or bad would it be to do what Dan did if it happened very far from here? Please show me on this line. [Show subjects standard severity judgment scale. Be sure to record number from scale below.]  [Response:] _________ [record number from scale; If the participant indicates an intermediate point on the scale, record the nearest whole number value.] |
| --- | --- |

# [#4: MARKET CHEATING]

In this other American community, one day a stranger comes to buy food from Sam in a farmer’s market. The stranger is not a local person and does not speak English. The stranger comes from Laos, a country that is very far from here. The stranger buys some fresh food from Sam and pays Sam the amount agreed. However, Sam does not give the stranger the fresh food that the stranger had chosen. Instead, he replaces the fresh food that the stranger had chosen with older food that is beginning to have a rotten smell. He wraps the food in a package so that the stranger is not aware of the switch until he is far away.

# [#4: MARKET CHEATING: Comprehension Questions]

[RA: Read the following to the subject]

“Because it is important that I make myself clear, I’d like to make sure that you understood me. Could you please tell me what happened in the story I just told you?”

|  | **Concept** | **Mentioned in P’s 1^st^ Summary? P1** | **Question if Not mentioned** | **Correct? R1** | **Correct? R2** |
| --- | --- | --- | --- | --- | --- |
| CC1 | Non Local |  | Was the stranger American? |  |  |
| CC2 | Received purchased goods |  | Did the stranger get the goods that he paid for? |  |  |
| CC3 | Time delay |  | When did the stranger become aware of the switch? |  |  |

[RA: If the participant gets one or more of the probes wrong (R1), say "Perhaps I did not make myself clear. Please let me read the story to you again;” then return to the story and re-read it once more. Then, go through the probes that the subject got wrong again, recording the responses in the R2 column (1 for a correct answer, 0 for an incorrect answer). After round 2, regardless of whether they still make mistakes, proceed to questions below.]

# [#4: MARKET CHEATING: Questions]

| [Severity] | In your personal view, how good or bad is what Sam did? Please show me on this line. [Show subjects standard severity judgment scale. Be sure to record number from scale below.]  [Response:] _________ [record number from scale; If the participant indicates an intermediate point on the scale, record the nearest whole number value.] |
| --- | --- |

| [Justification] | Why? [Record notes on participant’s response here:] |
| --- | --- |

| [Reputation] | What would people in his community think of Sam? Would they think he is a good person or a bad person? Please show me on this line. [Show subjects standard severity judgment scale. Be sure to record number from scale below.]  [Response:] _________ [record number from scale; If the participant indicates an intermediate point on the scale, record the nearest whole number value.] |
| --- | --- |

| [Long Ago] | What if this happened a long, long time ago, before your grandparents were born, even before their grandparents were born. In your personal view, how good or bad would it be to do what Sam did a very long time ago? Please show me on this line. [Show subjects standard severity judgment scale. Be sure to record number from scale below.]  [Response:] _________ [record number from scale; If the participant indicates an intermediate point on the scale, record the nearest whole number value.] |
| --- | --- |

| [Far Away] | What if this happened in place very far from here, a place that no one in this town has ever visited, and I have never visited either. In your personal view, how good or bad would it be to do what Sam did if it happened very far from here? Please show me on this line. [Show subjects standard severity judgment scale. Be sure to record number from scale below.]  [Response:] _________ [record number from scale; If the participant indicates an intermediate point on the scale, record the nearest whole number value.] |
| --- | --- |

| [Authority Dependence] | Suppose that the community leaders said that it is not bad for a man cheat another man in the market if the other man is not local and does not speak English. In your personal view, how good or bad would it be to do what Sam did, if the community leaders said that it was not bad? Please show me on this line.  [Show subjects standard severity judgment scale. Be sure to record number from scale below.]  [Response:] _________ [record number from scale; If the participant indicates an intermediate point on the scale, record the nearest whole number value.] |
| --- | --- |

# [#5: DEFAMATION]

In this other American community, Mark and David are neighbors. However, they do not like each other. One day, Mark decides to spread a false rumor that David has been stealing from others in the community. Though the rumor is not true, many people in the town believe it and it does great damage to David’s reputation.

# [#5: DEFAMATION: Comprehension Questions]

[RA: Read the following to the subject]

“Because it is important that I make myself clear, I’d like to make sure that you understood me. Could you please tell me what happened in the story I just told you?”

|  | **Concept** | **Mentioned in P’s 1^st^ Summary? P1** | **Question if Not mentioned** | **Correct? R1** | **Correct? R2** |
| --- | --- | --- | --- | --- | --- |
| DC1 | Disliked each other |  | Did Mark and David like each other, or dislike each other? |  |  |
| DC2 | False Rumor |  | Was the rumor about David true? |  |  |
| DC3 | Damage to Reputation |  | What happened to David’s reputation? |  |  |

[RA: If the participant gets one or more of the probes wrong (R1), say "Perhaps I did not make myself clear. Please let me read the story to you again;” then return to the story and re-read it once more. Then, go through the probes that the subject got wrong again, recording the responses in the R2 column (1 for a correct answer, 0 for an incorrect answer). After round 2, regardless of whether they still make mistakes, proceed to questions below.]

# [#5: DEFAMATION: Questions]

| [Severity] | In your personal view, how good or bad is what Mark did? Please show me on this line. [Show subjects standard severity judgment scale. Be sure to record number from scale below.]  [Response:] _________ [record number from scale; If the participant indicates an intermediate point on the scale, record the nearest whole number value.] |
| --- | --- |

| [Justification] | Why? [Record notes on participant’s response here:] |
| --- | --- |

| [Reputation] | What would people in his community think of Mark? Would they think he is a good person or a bad person? Please show me on this line. [Show subjects standard severity judgment scale. Be sure to record number from scale below.]  [Response:] _________ [record number from scale; If the participant indicates an intermediate point on the scale, record the nearest whole number value.] |
| --- | --- |

| [Authority Dependence] | Suppose that the community leaders said that it is not bad for a man to spread a false rumor about someone he does not like. In your personal view, how good or bad would it be to do what Mark did, if the community leaders said that it was not bad? Please show me on this line.  [Show subjects standard severity judgment scale. Be sure to record number from scale below.]  [Response:] _________ [record number from scale; If the participant indicates an intermediate point on the scale, record the nearest whole number value.] |
| --- | --- |

| [Long Ago] | What if this happened a long, long time ago, before your grandparents were born, even before their grandparents were born. In your personal view, how good or bad would it be to do what Mark did a very long time ago? Please show me on this line. [Show subjects standard severity judgment scale. Be sure to record number from scale below.]  [Response:] _________ [record number from scale; If the participant indicates an intermediate point on the scale, record the nearest whole number value.] |
| --- | --- |

| [Far Away] | What if this happened in place very far from here, a place that no one in this town has ever visited, and I have never visited either. In your personal view, how good or bad would it be to do what Mark did if it happened very far from here? Please show me on this line. [Show subjects standard severity judgment scale. Be sure to record number from scale below.]  [Response:] _________ [record number from scale; If the participant indicates an intermediate point on the scale, record the nearest whole number value.] |
| --- | --- |

# [#6: INJUSTICE]

Tom and Derrick are young men from this other American community. One night they have an argument and Tom starts a fight with Derrick and seriously injures him. A few days later, there is a community meeting to discuss what should be done about the incident. Before the meeting, Richard, an influential man in the community, accepts money from Tom as a bribe. As a result, Richard lies in the meeting and says that Derrick started the fight and everyone believes him. And so the community decides to punish Derrick but not to punish Tom.

# [#6: INJUSTICE: Comprehension Questions]

[RA: Read the following to the subject]

“Because it is important that I make myself clear, I’d like to make sure that you understood me. Could you please tell me what happened in the story I just told you?”

|  | **Concept** | **Mentioned in P’s 1^st^ Summary? P1** | **Question if Not mentioned** | **Correct? R1** | **Correct? R2** |
| --- | --- | --- | --- | --- | --- |
| IC1 | Tom starts fight |  | Who started the fight? |  |  |
| IC2 | Richard lies |  | Did Richard tell the truth about who started the fight? |  |  |
| IC3 | Tom bribed Richard |  | Why did Richard lie for Tom? |  |  |

[RA: If the participant gets one or more of the probes wrong (R1), say "Perhaps I did not make myself clear. Please let me read the story to you again;” then return to the story and re-read it once more. Then, go through the probes that the subject got wrong again, recording the responses in the R2 column (1 for a correct answer, 0 for an incorrect answer). After round 2, regardless of whether they still make mistakes, proceed to questions below.]

# [#6: INJUSTICE: Questions]

| [Severity] | In your personal view, how good or bad is what Richard did? Please show me on this line. [Show subjects standard severity judgment scale. Be sure to record number from scale below.]  [Response:] _________ [record number from scale; If the participant indicates an intermediate point on the scale, record the nearest whole number value.] |
| --- | --- |

| [Justification] | Why? [Record notes on participant’s response here:] |
| --- | --- |

| [Reputation] | What would people in his community think of Richard if they knew what he had done? Would they think he is a good person or a bad person? Please show me on this line. [Show subjects standard severity judgment scale. Be sure to record number from scale below.]  [Response:] _________ [record number from scale; If the participant indicates an intermediate point on the scale, record the nearest whole number value.] |
| --- | --- |

| [Long Ago] | What if this happened a long, long time ago, before your grandparents were born, even before their grandparents were born. In your personal view, how good or bad would it be to do what Richard did a very long time ago? Please show me on this line. [Show subjects standard severity judgment scale. Be sure to record number from scale below.]  [Response:] _________ [record number from scale; If the participant indicates an intermediate point on the scale, record the nearest whole number value.] |
| --- | --- |

| [Far Away] | What if this happened in place very far from here, a place that no one in this town has ever visited, and I have never visited either. In your personal view, how good or bad would it be to do what Richard did if it happened very far from here? Please show me on this line. [Show subjects standard severity judgment scale. Be sure to record number from scale below.]  [Response:] _________ [record number from scale; If the participant indicates an intermediate point on the scale, record the nearest whole number value.] |
| --- | --- |

| [Authority Dependence] | Suppose that the community leaders said that it is not bad for an influential person to speak in defense of an accused person in exchange for a bribe. In your personal view, how good or bad would it be to do what Richard did, if the community leaders said that it was not bad? Please show me on this line.  [Show subjects standard severity judgment scale. Be sure to record number from scale below.]  [Response:] _________ [record number from scale; If the participant indicates an intermediate point on the scale, record the nearest whole number value.] |
| --- | --- |

**CAUSAL ATTRIBUTION**

**[Preamble to Participants]**

Now, I would like to briefly remind you about some of the scenarios you just heard about, and ask you a few more questions about each story.

# [#1: STEALING FROM A STRANGER]

James is a man from another American community. While camping near the town, James encounters a stranger from Tuva, a country that is very far away from here. The stranger does not speak English. After the stranger passes James, the stranger puts his sack down and walks down a small hill to wash in a stream. When the stranger is out of sight, James opens his sack and looks at the contents. He finds $700, takes the money and walks away quickly. The stranger does not realize his money has been taken until he is back home in his country, and he is then too far away to do anything about it.

Now, please take a moment to think about all of the factors of the situation that might have led to the money being taken. For example, think about recent events in James’ life that might have made James want to take the money. Also, consider the fact that the stranger left his sack unattended. I will pause for a few seconds while you think about all of the causes that might have led to the money being taken.

[Pause for 10 seconds.]

- [**Q1 PERSON**] Now, how much do you feel that the money was taken because of the kind of person that James is, in comparison to all of the other causes?

Please answer using this scale (show *Person Scale*):

[Response:] _________

- [**Q2** **ANYONE**] Now, how much do you feel that other people would have been likely to take the money if they found themselves in the exact same situation?

Please answer using this scale (show *Anyone Scale*):

[Response:] _________

# Now I will remind you of another situation.

# [#2: BATTERY]

In this other American community, Stephen returns home feeling very angry because his belongings have been damaged in a storm. His wife, who he knows was not responsible for the damage to his belongings, greets him warmly as he enters the house. Because he is angry, he slaps her face very hard, causing her nose to bleed.

Now, please take a moment to think about all of the factors of the situation that might have led to the wife getting slapped. For example, think about recent events in Stephen’s life that might have made Stephen want to slap his wife. Also, consider the fact that the storm damaged Stephen’s belongings. I will pause for a few seconds while you think about all of the causes that might have led to the wife getting slapped.

[Pause for 10 seconds.]

- [**Q1** **ANYONE**] Now, how much do you feel that other people would have been likely to slap their wife if they found themselves in the exact same situation?

Please answer using this scale (show *Anyone Scale*):

[Response:] _________

- [**Q2 PERSON**] Now, how much do you feel that the wife was slapped because of the kind of person that Stephen is, in comparison to all of the other causes?

Please answer using this scale (show *Person Scale*):

[Response:] _________

# Now I will remind you of another situation.

# [#3: UNINTENTIONAL HARM]

In this other American community, Roger and Dan are walking on a very muddy path. Roger slips in the mud and accidentally knocks Dan down as he tries to regain his balance. Dan injures his arm in the fall. Dan knows that it was an accident. When he gets up Dan is very angry and hits Roger in the face.

Now, please take a moment to think about all of the factors of the situation that might have led to Roger getting hit. For example, think about recent events in Dan’s life that might have made Dan want to hit Roger. Also, consider the fact that Roger slipped and knocked Dan down. I will pause for a few seconds while you think about all of the causes that might have led to Roger getting hit.

[Pause for 10 seconds.]

- [**Q1 PERSON**] Now, how much do you feel that Roger was hit because of the kind of person that Dan is, in comparison to all of the other causes?

Please answer using this scale (show *Person Scale*):

[Response:] _________

- [**Q2** **ANYONE**] Now, how much do you feel that other people would have been likely to hit Roger if they found themselves in the exact same situation?

Please answer using this scale (show *Anyone Scale*):

[Response:] _________

# Now I will remind you of another situation.

# [#4: MARKET CHEATING]

In this other American community, one day a stranger comes to buy food from Sam in a farmer’s market. The stranger is not a local person and does not speak English. The stranger comes from Laos, a country that is very far from here. The stranger buys some fresh food from Sam and pays Sam the amount agreed. However, Sam does not give the stranger the fresh food that the stranger had chosen. Instead, he replaces the fresh food that the stranger had chosen with older food that is beginning to have a rotten smell. He wraps the food in a package so that the stranger is not aware of the switch until he is far away.

Now, please take a moment to think about all of the factors of the situation that might have led to the food getting switched. For example, think about recent events in Sam’s life that might have made Sam want to switch the food. Also, consider the fact that the stranger does not watch while the food is being wrapped up. I will pause for a few seconds while you think about all of the causes that might have led to the food getting switched.

[Pause for 10 seconds.]

- [**Q1** **ANYONE**] Now, how much do you feel that other people would have been likely to switch the food if they found themselves in the exact same situation?

Please answer using this scale (show *Anyone Scale*):

[Response:] _________

- [**Q2 PERSON**] Now, how much do you feel that the food was switched because of the kind of person that Sam is, in comparison to all of the other causes?

Please answer using this scale (show *Person Scale*):

[Response:] _________

# Now I will remind you of another situation.

# [#5: DEFAMATION]

In this other American community, Mark and David are neighbors. However, they do not like each other. One day, Mark decides to spread a false rumor that David has been stealing from others in the community. Though the rumor is not true, many people in the town believe it and it does great damage to David’s reputation.

Now, please take a moment to think about all of the factors of the situation that might have led to the rumor getting spread. For example, think about recent events in Mark’s life that might have made Mark want to spread the rumor. Also, consider the fact that Mark and David both dislike each other. I will pause for a few seconds while you think about all of the causes that might have led to the rumor getting spread.

[Pause for 10 seconds.]

- [**Q1 PERSON**] Now, how much do you feel that the rumor was spread because of the kind of person that Mark is, in comparison to all of the other causes?

Please answer using this scale (show *Person Scale*):

[Response:] _________

- [**Q2** **ANYONE**] Now, how much do you feel that other people would have been likely to spread the rumor if they found themselves in the exact same situation?

Please answer using this scale (show *Anyone Scale*):

[Response:] _________

# Now I will remind you of another situation.

# [#6: INJUSTICE]

Tom and Derrick are young men from this other American community. One night they have an argument and Tom starts a fight with Derrick and seriously injures him. A few days later, there is a community meeting to discuss what should be done about the incident. Before the meeting, Richard, an influential man in the community, accepts money from Tom as a bribe. As a result, Richard lies in the meeting and says that Derrick started the fight and everyone believes him. And so the community decides to punish Derrick but not to punish Tom.

Now, please take a moment to think about all of the factors of the situation that might have led to the lie about who started the fight being told. For example, think about recent events in Richard’s life that might have made Richard want to accept the bribe to tell the lie. Also, consider the possible explanations for why Tom and Derrick had an argument. I will pause for a few seconds while you think about all of the causes that might have led to the lie about who started the fight being told.

[Pause for 10 seconds.]

- [**Q1** **ANYONE**] Now, how much do you feel that other people would have been likely to tell the lie about who started the fight if they found themselves in the exact same situation?

Please answer using this scale (show *Anyone Scale*):

[Response:] _________

- [**Q2 PERSON**] Now, how much do you feel that the lie about who started the fight was told because of the kind of person that Richard is, in comparison to all of the other causes?

Please answer using this scale (show *Person Scale*):

[Response:] _________

Thanks, that is the end of the survey! Now we just have a few more questions about you.

[Go on to demographics.]

# [DEMOGRAPHICS]

Thank you for your participation in this survey. I just have a few more questions about you.

*Sex*: ________ *Age:* ________ *Ethnicity:* _______________ *Country of Birth:* ____________

*Current citizenship:*

- American
- Other
- Prefer not to Disclose

*Highest level of education personally achieved:* _____________________________

*Highest level of education achieved by a parent:* ______________________________

*Annual household income:* ______________________________

*How would describe your political views?*

- Very Liberal
- Slightly Liberal
- Moderate
- Slightly Conservative
- Very Conservative

Notes (concerns about distractions, interruptions, participant comprehension, sincerity, etc.):

______________________________________________________________________________________________________________________________________________________________________________________________________________________________________________________________________________________________________________________________________________________________________________________________________

# [DEBRIEFING]

Thanks again for your participation in this study. The researchers who designed it are interested in learning about people’s judgments of right and wrong, and whether these judgments are influenced by contexts such as where or when the action took place.

Thanks again!

[Pay participant.]

# Seoul STUDY PROTOCOL, ORDER VERSION 1

[괄호 안에 있는 내용들은 참가자들에게 읽는 게 아니고, RA를 위한 내용입니다.]

**각각의 시나리오에서의 이해 테스트를 위한 지시**

[RA: 참여자의 응답을 듣고, 참여자의 최초의 이야기에 대한 요약을 들으며 3개의 개념들 중 어떤 개념이 있다면 빈칸에 1을 표시하시오. 최초의 요약에 나오지 않은 각각의 아이템에 대해서 빈칸에 0을 써넣고, 상응하는 질문을 하시오, 그리고 나서 참여자가 질문에 대해서 알맞게 대답했는지 기록하시오; 맞는다면 1을 써넣고, 틀리다면 0을 써넣으시오.]

[RA: 만약 그 참여자가 한가지 또는 두 가지 질문에 대해서 틀리게 답했다면, "제가 명확하게 전달하지 못한 것 같습니다. 한번 더 이야기를 읽어드려도 될까요."하고 이야기로 돌아가서, 다시 한번 이야기를 읽으시오. 그리고 나서, 참여자가 틀리게 답했던 질문에 대해서 다시 한번 질문을 하면서 맞는다면 1을, 틀리다면 0을 표시하시오. 두 번 하고 나서, 참여자들이 실수를 하는 것에 상관없이, 밑의 질문을 하시오.]

**참가자들을 위한 서두**

이제부터 제가, 한국에서 일어난 사건들에 대한 이야기들을 읽어드리겠습니다. 그리고 각각의 이야기에 대한 질문들을 하겠습니다. 30분정도 소요될 예정이며, 마치시면 만원을 받게 되실 겁니다. 참여 여부는 완전히 참여자분의 의사에 따라서 하시면 됩니다. 그리고 만약 지금 시간이 충분하지 않으시다면 참여하지 않으셔도 되고, 또는 다른 날로 스케줄을 잡아드릴 수 있습니다. 시작하고 나서 만약 참여하길 원하지 않는다고 판단하신다면, 참여자 분께서 원하실 때 언제든 그만두실 수 있습니다. 참여하시겠습니까?

[RA: 참여자가 “알겠다”고 하면, datasheet1부터 채워넣기 시작함]

“앞으로 듣게되실 6개의 시나리오에 대한 질문들은 반복되는 형식이라 계속 듣는게 불필요하다고 느끼실 수 있을 것입니다. 하지만 그것 또한 연구의 일부이기 때문에 반복되는 질문들을 끝까지 듣고 답해주시길 바랍니다.”

# [#1: 외국인의 돈을 절도]

민준은 우리나라 다른 지방의 사람입니다. 근교에서 캠핑을 하다가 아이슬란드에서 온 외국인을 만났습니다. 그 외국인은 한국말을 할 줄 모릅니다. 외국인이 민준을 지나치고 나서, 가방을 내려놓고, 하천에서 손과 발을 씻기 위해 작은 언덕을 걸어 내려갔습니다. 그가 사라지고 나서, 민준은 그의 가방을 열고, 내용물을 봤습니다. 민준은 70만원을 발견했고, 돈을 가지고 빨리 그 자리를 떠나버렸습니다. 외국인은 아이슬란드로 돌아가기 전까지 자신의 돈을 누군가 가져갔다는 것을 알아차리지 못했고, 무언가를 하기에는 이미 늦었었습니다.

# [#1: 외국인의 돈을 절도: 이해 테스트]

[RA: 다음을 참여자에게 읽으시오.]

“이야기가 잘 전달되었는지 명확히 하는 것이 중요하기 때문에, 참여자 분께서 제 이야기를 이해하셨는지를 확인하고자 합니다. 제가 방금 말씀 드린 이야기에서 어떤 일이 일어났었는지 이야기해주실 수 있나요?”

| **개념** | **참여자의 최초의 요약에서 언급되었나요?** | **만약에 언급되지 않았다면 물으시오** | **맞았나요?** | **맞았나요?** |
| --- | --- | --- | --- | --- |
| 외국인의 것을 절도함 |  | 민준이 누구의 것을 절도하였나? |  |  |
| 다른 민족인 외국인 |  | 절도 당한 사람이 한국인이었나요? |  |  |
| 도난 당했다는 것을 늦게 알아차림 |  | 외국인은 한국에 있을 때 자신이 돈을 도난 당했다는 것을 알아차렸나요? |  |  |

 [RA: 만약 그 참여자가 한가지 또는 두 가지 질문에 대해서 틀리게 답했다면, "아마도 제가 명확하게 전달하지 못한 것 같습니다. 한번 더 제가 이야기를 읽어드려도 될까요."하고 이야기로 돌아가서, 다시 한번 이야기를 읽으시오. 그리고 나서, 참여자가 틀리게 답했던 질문에 대해서 다시 한번 질문을 하면서 맞는다면 1을, 틀리다면 0을 표시하시오. 두 번 하고 나서, 참여자들이 실수를 하는 것에 상관없이, 밑의 질문을 하시오.]

# [#1: 외국인의 돈을 절도: 질문들]

| [심각성] | 개인적으로 생각하시기에, 민준이 한 행위는 얼마나 좋은 행위거나 나쁜 행위입니까? 여기에 표시해주십시오. [참여자들에게, 심각성 판단 척도를 보여주고, 아래에 응답을 기록하시오.]  [응답:] _________ [참여자가 가리킨 척도의 숫자를 기록하시오; 만약 참여자가 척도의 숫자들이 아닌 숫자와 숫자 사이 어딘가를 가리켰다면, 그것에서 가장 가까운 숫자를 적으시오.] |
| --- | --- |

| [이유] | 그렇게 생각한 까닭은 무엇입니까? [참여자의 응답을 적으시오:] |
| --- | --- |

| [평판/명성] | 민준과 같은 공동체에 속한 사람들이 민준에 대해서 어떻게 생각할 것 같습니까? 사람들이 그를 좋은 사람이라고 생각할 것 같습니까 아니면 나쁜 사람이라고 생각할 것 같습니까? 이 척도를 가리키며 말씀해주십시오. [참여자들에게 심각성 판단 척도를 보여주고, 아래에 응답을 기록하시오.]  [응답:] _________ [참여자가 가리킨 척도의 숫자를 기록하시오; 만약 참여자가 척도의 숫자들이 아닌 숫자와 숫자 사이 어딘가를 가리켰다면, 그것에서 가장 가까운 숫자를 적으시오.] |
| --- | --- |

| [권위자에의 의존] | 어떤 공동체의 리더들이 내가 사는 곳의 근처에 살지 않거나 한국말을 하지 못하는 사람의 물건이나 돈을 훔치는 행위는 나쁘지 않다고 말했다고 해봅시다. 만약에 공동체의 리더들이 그렇게 말했다면, 개인적으로 생각하시기에, 민준이 한 행위가 얼만큼 좋거나 나쁘다고 생각하십니까? 이 척도를 가리키며 말씀해주십시오. [참여자들에게 심각성 판단 척도를 보여주고, 아래에 응답을 기록하시오.]  [응답:] _________ [참여자가 가리킨 척도의 숫자를 기록하시오; 만약 참여자가 척도의 숫자들이 아닌 숫자와 숫자 사이 어딘가를 가리켰다면, 그것에서 가장 가까운 숫자를 적으시오.] |
| --- | --- |

| [먼 옛날] | 만약에 이 일이 아주 아주 먼 옛날, 당신의 조부모가 태어나기도 전에, 심지어 당신의 조부모의 조부모가 태어나기도 전에 일어난 일이라면, 개인적으로 생각하시기에, 민준이 한 행위가 얼만큼 좋거나 나쁘다고 생각하십니까? 이 척도를 가리키며 말씀해주십시오. [참여자들에게 심각성 판단 척도를 보여주고, 아래에 응답을 기록하시오.]  [응답:] _________ [참여자가 가리킨 척도의 숫자를 기록하시오; 만약 참여자가 척도의 숫자들이 아닌 숫자와 숫자 사이 어딘가를 가리켰다면, 그것에서 가장 가까운 숫자를 적으시오.] |
| --- | --- |

| [먼 장소] | 만약에 여기서 매우 멀리 떨어진 곳에서 이 일이 일어났다고 해봅시다. 그 멀리 떨어진 곳은 우리 나라의 사람들 중 누구도 가본적이 없는 곳이며, 저 또한 한번도 가본적이 없는 곳입니다. 그렇다면, 개인적으로 생각하시기에, 민준이 한 행위가 얼만큼 좋거나 나쁘다고 생각하십니까? 이 척도를 가리키며 말씀해주십시오. [참여자들에게 심각성 판단 척도를 보여주고, 아래에 응답을 기록하시오.]  [응답:] _________ [참여자가 가리킨 척도의 숫자를 기록하시오; 만약 참여자가 척도의 숫자들이 아닌 숫자와 숫자 사이 어딘가를 가리켰다면, 그것에서 가장 가까운 숫자를 적으시오.] |
| --- | --- |

# [#2: 구타]

이 일의 배경은 서울입니다. 준서는 그의 소지품들이 폭풍우 때문에 망가졌다는 것을 알고 집에 매우 화난 채로 돌아왔습니다. 준서는 아내가 소지품들이 망가진 것에 아무런 책임이 없다는 것을 알았음에도 불구하고 아내가 따뜻하게 인사하며 그를 맞이했을 때 단지 화가 났다는 이유만으로 아내의 얼굴을 세게 때렸고, 아내의 코에서 코피가 흘렀습니다.

# [#2: 구타: 이해 테스트]

[RA: 다음을 참여자에게 읽으시오.]

“이야기가 잘 전달되었는지 명확히 하는 것이 중요하기 때문에, 참여자 분께서 제 이야기를 이해하셨는지를 확인하고자 합니다. 제가 방금 말씀 드린 이야기에서 어떤 일이 일어났었는지 이야기해주실 수 있나요?”

| **개념** | **참여자의 최초의 요약에서 언급되었나요?** | **만약에 언급되지 않았다면 물으시오** | **맞았나요?** | **맞았나요?** |
| --- | --- | --- | --- | --- |
| 소지품들이 망가짐 |  | 준서의 소지품들에 어떤 일이 일어났나요? |  |  |
| 아내는 책임이 없음 |  | 준서의 아내가 준서의 소지품들의 훼손에 책임이 있었나요? |  |  |
| 아내를 때림 |  | 준서가 아내에게 무엇을 했나요? |  |  |

 [RA: 만약 그 참여자가 한가지 또는 두 가지 질문에 대해서 틀리게 답했다면, "아마도 제가 명확하게 전달하지 못한 것 같습니다. 한번 더 제가 이야기를 읽어드려도 될까요."하고 이야기로 돌아가서, 다시 한번 이야기를 읽으시오. 그리고 나서, 참여자가 틀리게 답했던 질문에 대해서 다시 한번 질문을 하면서 맞는다면 1을, 틀리다면 0을 표시하시오. 두 번 하고 나서, 참여자들이 실수를 하는 것에 상관없이, 밑의 질문을 하시오.]

# [#2: 구타: 질문들]

| [심각성] | 개인적으로 생각하시기에, 준서가 한 행위는 얼마나 좋은 행위거나 나쁜 행위입니까? 여기에 표시해주십시오. [참여자들에게 심각성 판단 척도를 보여주고, 아래에 응답을 기록하시오.]  [응답:] _________ [참여자가 가리킨 척도의 숫자를 기록하시오; 만약 참여자가 척도의 숫자들이 아닌 숫자와 숫자 사이 어딘가를 가리켰다면, 그것에서 가장 가까운 숫자를 적으시오.] |
| --- | --- |

| [이유] | 그렇게 생각한 까닭은 무엇입니까? [참여자의 응답을 적으시오:] |
| --- | --- |

| [평판/명성] | 준서와 같은 공동체에 속한 사람들이 준서에 대해서 어떻게 생각할 것 같습니까? 사람들이 그를 좋은 사람이라고 생각할 것 같습니까 아니면 나쁜 사람이라고 생각할 것 같습니까? 이 척도를 가리키며 말씀해주십시오. [참여자들에게 심각성 판단 척도를 보여주고, 아래에 응답을 기록하시오.]  [응답:] _________ [참여자가 가리킨 척도의 숫자를 기록하시오; 만약 참여자가 척도의 숫자들이 아닌 숫자와 숫자 사이 어딘가를 가리켰다면, 그것에서 가장 가까운 숫자를 적으시오.] |
| --- | --- |

| [먼 옛날] | 만약에 이 일이 아주 아주 먼 옛날, 당신의 조부모가 태어나기도 전에, 심지어 당신의 조부모의 조부모가 태어나기도 전에 일어난 일이라면, 개인적으로 생각하시기에, 준서가 한 행위가 얼만큼 좋거나 나쁘다고 생각하십니까? 이 척도를 가리키며 말씀해주십시오. [참여자들에게 심각성 판단 척도를 보여주고, 아래에 응답을 기록하시오.]  [응답:] _________ [참여자가 가리킨 척도의 숫자를 기록하시오; 만약 참여자가 척도의 숫자들이 아닌 숫자와 숫자 사이 어딘가를 가리켰다면, 그것에서 가장 가까운 숫자를 적으시오.] |
| --- | --- |

| [먼 장소] | 만약에 여기서 매우 멀리 떨어진 곳에서 이 일이 일어났다고 해봅시다. 그 멀리 떨어진 곳은 우리 나라의 사람들 중 누구도 가본적이 없는 곳이며, 저 또한 한번도 가본적이 없는 곳입니다. 그렇다면, 개인적으로 생각하시기에, 준서가 한 행위가 얼만큼 좋거나 나쁘다고 생각하십니까? 이 척도를 가리키며 말씀해주십시오. [참여자들에게 심각성 판단 척도를 보여주고, 아래에 응답을 기록하시오.]  [응답:] _________ [참여자가 가리킨 척도의 숫자를 기록하시오; 만약 참여자가 척도의 숫자들이 아닌 숫자와 숫자 사이 어딘가를 가리켰다면, 그것에서 가장 가까운 숫자를 적으시오.] |
| --- | --- |

| [권위자에의 의존] | 어떤 공동체의 리더들이, 만약에 누군가 화가 났다면 아내를 때리는 행위는 나쁘지 않다고 말했다고 해봅시다. 만약에 공동체의 리더들이 그렇게 말했다면, 개인적으로 생각하시기에, 준서가 한 행위가 얼만큼 좋거나 나쁘다고 생각하십니까? 이 척도를 가리키며 말씀해주십시오. [참여자들에게 심각성 판단 척도를 보여주고, 아래에 응답을 기록하시오.]  [응답:] _________ [참여자가 가리킨 척도의 숫자를 기록하시오; 만약 참여자가 척도의 숫자들이 아닌 숫자와 숫자 사이 어딘가를 가리켰다면, 그것에서 가장 가까운 숫자를 적으시오.] |
| --- | --- |

# [#3: 의도하지 않은 피해]

이 일의 배경도 역시 서울입니다. 현준과 승민은 진흙투성이인 길을 따라서 걷고 있었습니다. 현준은 진흙 속에서 미끄러졌고, 다시 균형을 잡는 와중에 의도치 않게 승민을 쳤습니다. 승민이 넘어지면서 팔을 다치게 되었습니다. 승민은 현준이 의도한 게 아니라는 걸 알고 있었습니다. 그럼에도 굉장히 화가 나서 승민이 일어나고서는 현준의 얼굴을 때렸습니다.

# [#3: 의도하지 않은 피해: 이해 테스트]

[RA: 다음을 참여자에게 읽으시오.]

“이야기가 잘 전달되었는지 명확히 하는 것이 중요하기 때문에, 참여자 분께서 제 이야기를 이해하셨는지를 확인하고자 합니다. 제가 방금 말씀 드린 이야기에서 어떤 일이 일어났었는지 이야기해주실 수 있나요?” “이야기가 잘 전달되었는지 명확히 하는 것이 중요하기 때문에, 참여자 분께서 제 이야기를 이해하셨는지를 확인하고자 합니다. 제가 방금 말씀 드린 이야기에서 어떤 일이 일어났었는지 이야기해주실 수 있나요?”

| **개념** | **참여자의 최초의 요약에서 언급되었나요?** | **만약에 언급되지 않았다면 물으시오** | **맞았나요?** | **맞았나요?** |
| --- | --- | --- | --- | --- |
| 의도 |  | 현준이 승민을 의도적으로 쳤나요? |  |  |
| 부상 |  | 승민이가 넘어지면서 다쳤나요? |  |  |
| 신체적인 응징 |  | 승민이 일어나고 나서 현준에게 무엇을 했나요? |  |  |

 [RA: 만약 그 참여자가 한가지 또는 두 가지 질문에 대해서 틀리게 답했다면, "아마도 제가 명확하게 전달하지 못한 것 같습니다. 한번 더 제가 이야기를 읽어드려도 될까요."하고 이야기로 돌아가서, 다시 한번 이야기를 읽으시오. 그리고 나서, 참여자가 틀리게 답했던 질문에 대해서 다시 한번 질문을 하면서 맞는다면 1을, 틀리다면 0을 표시하시오. 두 번 하고 나서, 참여자들이 실수를 하는 것에 상관없이, 밑의 질문을 하시오.]

# [#3: 의도하지 않은 피해: 질문들]

| [심각성] | 개인적으로 생각하시기에, 승민이 한 행위는 얼마나 좋은 행위거나 나쁜 행위입니까? 여기에 표시해주십시오. [참여자들에게 심각성 판단 척도를 보여주시오.]  [응답:] _________ [참여자가 가리킨 척도의 숫자를 기록하시오; 만약 참여자가 척도의 숫자들이 아닌 숫자와 숫자 사이 어딘가를 가리켰다면, 그것에서 가장 가까운 숫자를 적으시오.] |
| --- | --- |

| [이유] | 그렇게 생각한 까닭은 무엇입니까? [참여자의 응답을 적으시오:] |
| --- | --- |

| [평판/명성] | 승민과 같은 공동체에 속한 사람들이 승민에 대해서 어떻게 생각할 것 같습니까? 사람들이 그를 좋은 사람이라고 생각할 것 같습니까 아니면 나쁜 사람이라고 생각할 것 같습니까? 이 척도를 가리키며 말씀해주십시오. [참여자들에게 심각성 판단 척도를 보여주고, 아래에 응답을 기록하시오.]  [응답:] _________ [참여자가 가리킨 척도의 숫자를 기록하시오; 만약 참여자가 척도의 숫자들이 아닌 숫자와 숫자 사이 어딘가를 가리켰다면, 그것에서 가장 가까운 숫자를 적으시오.] |
| --- | --- |

| [권위자에의 의존] | 어떤 공동체의 리더들이, 만약에 의도치 않게 어떤 사람이 누군가에게 부상을 입혔다면, 다친 사람이 부상을 입힌 사람을 때리는 게 나쁘지 않다고 말했다고 해봅시다. 만약에 공동체의 리더들이 그렇게 말했다면, 개인적으로 생각하시기에, 승민이 한 행위가 얼만큼 좋거나 나쁘다고 생각하십니까? 이 척도를 가리키며 말씀해주십시오. [참여자들에게 심각성 판단 척도를 보여주고, 아래에 응답을 기록하시오.]  [응답:] _________ [참여자가 가리킨 척도의 숫자를 기록하시오; 만약 참여자가 척도의 숫자들이 아닌 숫자와 숫자 사이 어딘가를 가리켰다면, 그것에서 가장 가까운 숫자를 적으시오.] |
| --- | --- |

| [먼 옛날] | 만약에 이 일이 아주 아주 먼 옛날, 당신의 조부모가 태어나기도 전에, 심지어 당신의 조부모의 조부모가 태어나기도 전에 일어난 일이라면, 개인적으로 생각하시기에, 승민이 한 행위가 얼만큼 좋거나 나쁘다고 생각하십니까? 이 척도를 가리키며 말씀해주십시오. [참여자들에게 심각성 판단 척도를 보여주고, 아래에 응답을 기록하시오.]  [응답:] _________ [참여자가 가리킨 척도의 숫자를 기록하시오; 만약 참여자가 척도의 숫자들이 아닌 숫자와 숫자 사이 어딘가를 가리켰다면, 그것에서 가장 가까운 숫자를 적으시오.] |
| --- | --- |

| [먼 장소] | 만약에 여기서 매우 멀리 떨어진 곳에서 이 일이 일어났다고 해봅시다. 그 멀리 떨어진 곳은 우리 나라의 사람들 중 누구도 가본적이 없는 곳이며, 저 또한 한번도 가본적이 없는 곳입니다. 그렇다면, 개인적으로 생각하시기에, 승민이 한 행위가 얼만큼 좋거나 나쁘다고 생각하십니까? 이 척도를 가리키며 말씀해주십시오. [참여자들에게 심각성 판단 척도를 보여주고, 아래에 응답을 기록하시오.]  [응답:] _________ [참여자가 가리킨 척도의 숫자를 기록하시오; 만약 참여자가 척도의 숫자들이 아닌 숫자와 숫자 사이 어딘가를 가리켰다면, 그것에서 가장 가까운 숫자를 적으시오.] |
| --- | --- |

# [#4: 시장에서의 사기]

이 일의 배경도 역시 서울입니다. 어느 날 어떤 외국인이 광장시장에 음식을 사러 갔습니다. 그 외국인은 우리나라 사람이 아니고, 한국말을 할 줄 모릅니다. 그는 우리나라에서 굉장히 먼 핀란드에서 왔습니다. 외국인은 민성에게서 신선한 음식을 샀고, 음식에 대한 값을 지불했습니다. 하지만, 민성은 외국인에게, 그가 선택한 신선한 음식을 주지 않았습니다. 그가 고른 신선한 음식 대신 썩은 냄새가 나려고 하는 옛날 음식을 줬습니다. 민성은 외국인이 그 자리를 떠날 때까지 그 사실을 모르게 하기 위해서 음식을 잘 포장했습니다.

# [#4: MARKET CHEATING: 이해 테스트]

[RA: 다음을 참여자에게 읽으시오.]

“이야기가 잘 전달되었는지 명확히 하는 것이 중요하기 때문에, 참여자 분께서 제 이야기를 이해하셨는지를 확인하고자 합니다. 제가 방금 말씀 드린 이야기에서 어떤 일이 일어났었는지 이야기해주실 수 있나요?”

| **개념** | **참여자의 최초의 요약에서 언급되었나요?** | **만약에 언급되지 않았다면 물으시오** | **맞았나요?** | **맞았나요?** |
| --- | --- | --- | --- | --- |
| 이 지역사람이 아님 |  | 시장에서 음식을 산 사람이 한국사람이었나요? |  |  |
| 구매한 음식을 받음 |  | 그 외국인이 그가 지불한 음식들을 받았나요? |  |  |
| 뒤늦게 알게 됨 |  | 그 외국인이 음식이 바뀌었다는 것을 언제 알아차렸나요? |  |  |

 [RA: 만약 그 참여자가 한가지 또는 두 가지 질문에 대해서 틀리게 답했다면, "아마도 제가 명확하게 전달하지 못한 것 같습니다. 한번 더 제가 이야기를 읽어드려도 될까요."하고 이야기로 돌아가서, 다시 한번 이야기를 읽으시오. 그리고 나서, 참여자가 틀리게 답했던 질문에 대해서 다시 한번 질문을 하면서 맞는다면 1을, 틀리다면 0을 표시하시오. 두 번 하고 나서, 참여자들이 실수를 하는 것에 상관없이, 밑의 질문을 하시오.]

# [#4: 시장에서의 사기: 질문들]

| [심각성] | 개인적으로 생각하시기에, 민성이 한 행위는 얼마나 좋은 행위거나 나쁜 행위입니까? 여기에 표시해주십시오. [참여자들에게 심각성 판단 척도를 보여주고, 아래에 응답을 기록하시오.]  [응답:] _________ [참여자가 가리킨 척도의 숫자를 기록하시오; 만약 참여자가 척도의 숫자들이 아닌 숫자와 숫자 사이 어딘가를 가리켰다면, 그것에서 가장 가까운 숫자를 적으시오.] |
| --- | --- |

| [이유] | 그렇게 생각한 까닭은 무엇입니까? [참여자의 응답을 적으시오:] |
| --- | --- |

| [평판/명성] | 민성과 같은 공동체에 속한 사람들이 민성에 대해서 어떻게 생각할 것 같습니까? 사람들이 그를 좋은 사람이라고 생각할 것 같습니까 아니면 나쁜 사람이라고 생각할 것 같습니까? 이 척도를 가리키며 말씀해주십시오. [참여자들에게 심각성 판단 척도를 보여주시오]  [응답:] _________ [참여자가 가리킨 척도의 숫자를 기록하시오; 만약 참여자가 척도의 숫자들이 아닌 숫자와 숫자 사이 어딘가를 가리켰다면, 그것에서 가장 가까운 숫자를 적으시오.] |
| --- | --- |

| [먼 옛날] | 만약에 이 일이 아주 아주 먼 옛날, 당신의 조부모가 태어나기도 전에, 심지어 당신의 조부모의 조부모가 태어나기도 전에 일어난 일이라면, 개인적으로 생각하시기에, 민성이 한 행위가 얼만큼 좋거나 나쁘다고 생각하십니까? 이 척도를 가리키며 말씀해주십시오. [참여자들에게 심각성 판단 척도를 보여주고, 아래에 응답을 기록하시오.]  [응답:] _________ [참여자가 가리킨 척도의 숫자를 기록하시오; 만약 참여자가 척도의 숫자들이 아닌 숫자와 숫자 사이 어딘가를 가리켰다면, 그것에서 가장 가까운 숫자를 적으시오.] |
| --- | --- |

| [먼 장소] | 만약에 여기서 매우 멀리 떨어진 곳에서 이 일이 일어났다고 해봅시다. 그 멀리 떨어진 곳은 우리 나라의 사람들 중 누구도 가본적이 없는 곳이며, 저 또한 한번도 가본적이 없는 곳입니다. 그렇다면, 개인적으로 생각하시기에, 민성이 한 행위가 얼만큼 좋거나 나쁘다고 생각하십니까? 이 척도를 가리키며 말씀해주십시오. [참여자들에게 심각성 판단 척도를 보여주고, 아래에 응답을 기록하시오.]  [응답:] _________ [참여자가 가리킨 척도의 숫자를 기록하시오; 만약 참여자가 척도의 숫자들이 아닌 숫자와 숫자 사이 어딘가를 가리켰다면, 그것에서 가장 가까운 숫자를 적으시오.] |
| --- | --- |

| [권위자에의 의존] | 어떤 공동체의 리더들이 만약에 누군가가 서울 사람이 아니고, 한국말을 하지 못한다면 시장에서 그에게 사기를 치는 것이 나쁘지 않다고 했다고 해봅시다. 만약에 공동체의 리더들이 그렇게 말했다면, 개인적으로 생각하시기에, 민성이 한 행위가 얼만큼 좋거나 나쁘다고 생각하십니까? 이 척도를 가리키며 말씀해주십시오. [참여자들에게 표준이 되는 심각성 판단 척도를 보여주고, 아래에 응답을 기록하시오.]  [응답:] _________ [참여자가 가리킨 척도의 숫자를 기록하시오; 만약 참여자가 척도의 숫자들이 아닌 숫자와 숫자 사이 어딘가를 가리켰다면, 그것에서 가장 가까운 숫자를 적으시오.] |
| --- | --- |

# [#5: 명예훼손]

이 일의 배경도 역시 서울입니다. 동현과 성민은 이웃입니다. 하지만, 그들은 서로 좋아하지 않습니다. 어느 날, 동현은, 성민이 다른 사람의 물건 또는 돈을 훔쳤다는 거짓 소문을 퍼뜨렸습니다. 그 소문은 사실이 아님에도 불구하고, 많은 사람들은 그것을 믿었고, 성민은 자신의 평판에 치명적인 해를 입었습니다.

# [#5: 명예훼손: 이해 테스트]

[RA: 다음을 참여자에게 읽으시오.]

“이야기가 잘 전달되었는지 명확히 하는 것이 중요하기 때문에, 참여자 분께서 제 이야기를 이해하셨는지를 확인하고자 합니다. 제가 방금 말씀 드린 이야기에서 어떤 일이 일어났었는지 이야기해주실 수 있나요?”

| **개념** | **참여자의 최초의 요약에서 언급되었나요?** | **만약에 언급되지 않았다면 물으시오** | **맞았나요?** | **맞았나요?** |
| --- | --- | --- | --- | --- |
| 서로 싫어함 |  | 동현과 성민이 서로 좋아하는 사이였나요, 아니면 싫어하는 사이였나요? |  |  |
| 거짓 루머 |  | 성민에 대한 루머가 사실이었나요? |  |  |
| 평판에 해를 입힘 |  | 성민의 평판이 어떻게 되었나요? |  |  |

[RA: 만약 그 참여자가 한가지 또는 두 가지 질문에 대해서 틀리게 답했다면, "아마도 제가 명확하게 전달하지 못한 것 같습니다. 한번 더 제가 이야기를 읽어드려도 될까요."하고 이야기로 돌아가서, 다시 한번 이야기를 읽으시오. 그리고 나서, 참여자가 틀리게 답했던 질문에 대해서 다시 한번 질문을 하면서 맞는다면 1을, 틀리다면 0을 표시하시오. 두 번 하고 나서, 참여자들이 실수를 하는 것에 상관없이, 밑의 질문을 하시오.]

# [#5: 명예훼손: 질문들]

| [심각성] | 개인적으로 생각하시기에, 동현이 한 행위는 얼마나 좋은 행위거나 나쁜 행위입니까? 여기에 표시해주십시오. [참여자들에게 심각성 판단 척도를 보여주고, 아래에 응답을 기록하시오.]  [응답:] _________ [참여자가 가리킨 척도의 숫자를 기록하시오; 만약 참여자가 척도의 숫자들이 아닌 숫자와 숫자 사이 어딘가를 가리켰다면, 그것에서 가장 가까운 숫자를 적으시오.] |
| --- | --- |

| [이유] | 그렇게 생각한 까닭은 무엇입니까? [참여자의 응답을 적으시오:] |
| --- | --- |

| [평판/명성] | 동현과 같은 공동체에 속한 사람들이 그를 좋은 사람이라고 생각할 것 같습니까 아니면 나쁜 사람이라고 생각할 것 같습니까? 이 척도를 가리키며 말씀해주십시오. [참여자들에게 심각성 판단 척도를 보여주고, 아래에 응답을 기록하시오.]  [응답:] _________ [참여자가 가리킨 척도의 숫자를 기록하시오; 만약 참여자가 척도의 숫자들이 아닌 숫자와 숫자 사이 어딘가를 가리켰다면, 그것에서 가장 가까운 숫자를 적으시오.] |
| --- | --- |

| [권위자에의 의존] | 만약에 어떤 공동체의 리더들이 누군가 자신이 좋아하지 않는 사람에 대해서 거짓 루머를 퍼뜨리는 게 나쁘지 않다고 말했다고 해봅시다. 공동체의 리더들이 그렇게 말했다면, 개인적으로 생각하시기에, 동현이 한 행위가 얼만큼 좋거나 나쁘다고 생각하십니까? 이 척도를 가리키며 말씀해주십시오.  [참여자들에게 심각성 판단 척도를 보여주고, 아래에 응답을 기록하시오.]  [응답:] _________ [참여자가 가리킨 척도의 숫자를 기록하시오; 만약 참여자가 척도의 숫자들이 아닌 숫자와 숫자 사이 어딘가를 가리켰다면, 그것에서 가장 가까운 숫자를 적으시오.] |
| --- | --- |

| [먼 옛날] | 만약에 이 일이 아주 아주 먼 옛날, 당신의 조부모가 태어나기도 전에, 심지어 당신의 조부모의 조부모가 태어나기도 전에 일어난 일이라면, 개인적으로 생각하시기에, 동현이 한 행위가 얼만큼 좋거나 나쁘다고 생각하십니까? 이 척도를 가리키며 말씀해주십시오. [참여자들에게 심각성 판단 척도를 보여주고, 아래에 응답을 기록하시오.]  [응답:] _________ [참여자가 가리킨 척도의 숫자를 기록하시오; 만약 참여자가 척도의 숫자들이 아닌 숫자와 숫자 사이 어딘가를 가리켰다면, 그것에서 가장 가까운 숫자를 적으시오.] |
| --- | --- |

| [먼 장소] | 만약에 여기서 매우 멀리 떨어진 곳에서 이 일이 일어났다고 해봅시다. 그 멀리 떨어진 곳은 우리 나라의 사람들 중 누구도 가본적이 없는 곳이며, 저 또한 한번도 가본적이 없는 곳입니다. 그렇다면, 개인적으로 생각하시기에, 동현이 한 행위가 얼만큼 좋거나 나쁘다고 생각하십니까? 이 척도를 가리키며 말씀해주십시오. [참여자들에게 심각성 판단 척도를 보여주고, 아래에 응답을 기록하시오.]  [응답:] _________ [참여자가 가리킨 척도의 숫자를 기록하시오; 만약 참여자가 척도의 숫자들이 아닌 숫자와 숫자 사이 어딘가를 가리켰다면, 그것에서 가장 가까운 숫자를 적으시오.] |
| --- | --- |

# [#6: 부정의]

규태와 태경은 서울출신의 청년들입니다. 어느 날 밤에 그 둘은 언쟁을 했습니다. 규태가 먼저 태경과 싸움을 시작했고, 그에게 부상을 입혔습니다. 며칠이 지난 뒤에, 그 사건에 대해서 어떤 조치가 취해져야 하는지 논의하는 지역사회의 회의가 있었습니다. 회의 전에, 서울에서 영향력 있는 인물인 연우는 규태로부터 뇌물로 돈을 받았습니다. 연우는 회의에서 거짓말을 했고, 태경이 싸움을 시작했다고 말했습니다. 모두가 연우를 믿었고, 지역사회에서 규태가 아닌 태경을 처벌하기로 했습니다.

# [#6: 부정의: 이해 테스트]

[RA: 다음을 참여자에게 읽으시오.]

“이야기가 잘 전달되었는지 명확히 하는 것이 중요하기 때문에, 참여자 분께서 제 이야기를 이해하셨는지를 확인하고자 합니다. 제가 방금 말씀 드린 이야기에서 어떤 일이 일어났었는지 이야기해주실 수 있나요?”

| **개념** | **참여자의 최초의 요약에서 언급되었나요?** | **만약에 언급되지 않았다면 물으시오** | **맞았나요?** | **맞았나요?** |
| --- | --- | --- | --- | --- |
| 규태가 먼저 싸움을 시작했다 |  | 누가 먼저 싸움을 시작했나요? |  |  |
| 연우가 거짓말했다 |  | 연우가 누군가 먼저 싸움을 시작했는지에 대해 사실대로 말했나요? |  |  |
| 규태가 연우에게 뇌물을 줬다 |  | 왜 연우는 규태를 위해서 거짓말했나요? |  |  |

[RA: 만약 그 참여자가 한가지 또는 두 가지 질문에 대해서 틀리게 답했다면, "아마도 제가 명확하게 전달하지 못한 것 같습니다. 한번 더 제가 이야기를 읽어드려도 될까요."하고 이야기로 돌아가서, 다시 한번 이야기를 읽으시오. 그리고 나서, 참여자가 틀리게 답했던 질문에 대해서 다시 한번 질문을 하면서 맞는다면 1을, 틀리다면 0을 표시하시오. 두 번 하고 나서, 참여자들이 실수를 하는 것에 상관없이, 밑의 질문을 하시오.]

# [#6: 부정의: 질문들]

| [심각성] | 개인적으로 생각하시기에, 연우가 한 행위는 얼마나 좋은 행위거나 나쁜 행위입니까? 여기에 표시해주십시오. [참여자들에게 심각성 판단 척도를 보여주고, 아래에 응답을 기록하시오.]  [응답:] _________ [참여자가 가리킨 척도의 숫자를 기록하시오; 만약 참여자가 척도의 숫자들이 아닌 숫자와 숫자 사이 어딘가를 가리켰다면, 그것에서 가장 가까운 숫자를 적으시오.] |
| --- | --- |

| [이유] | 그렇게 생각한 까닭은 무엇입니까? [참여자의 응답을 적으시오:] |
| --- | --- |

| [평판/명성] | 연우와 같은 공동체에 속한 사람들이 연우에 대해서 어떻게 생각할 것 같습니까? 사람들이 그를 좋은 사람이라고 생각할 것 같습니까 아니면 나쁜 사람이라고 생각할 것 같습니까? 이 척도를 가리키며 말씀해주십시오. [참여자들에게 심각성 판단 척도를 보여주고, 아래에 응답을 기록하시오.]  [응답:] _________ [참여자가 가리킨 척도의 숫자를 기록하시오; 만약 참여자가 척도의 숫자들이 아닌 숫자와 숫자 사이 어딘가를 가리켰다면, 그것에서 가장 가까운 숫자를 적으시오.] |
| --- | --- |

| [먼 옛날] | 만약에 이 일이 아주 아주 먼 옛날, 당신의 조부모가 태어나기도 전에, 심지어 당신의 조부모의 조부모가 태어나기도 전에 일어난 일이라면, 개인적으로 생각하시기에, 연우가 한 행위가 얼만큼 좋거나 나쁘다고 생각하십니까? 이 척도를 가리키며 말씀해주십시오. [참여자들에게 심각성 판단 척도를 보여주고, 아래에 응답을 기록하시오.]  [응답:] _________ [참여자가 가리킨 척도의 숫자를 기록하시오; 만약 참여자가 척도의 숫자들이 아닌 숫자와 숫자 사이 어딘가를 가리켰다면, 그것에서 가장 가까운 숫자를 적으시오.] |
| --- | --- |

| [먼 장소] | 만약에 여기서 매우 멀리 떨어진 곳에서 이 일이 일어났다고 해봅시다. 그 멀리 떨어진 곳은 우리 나라의 사람들 중 누구도 가본적이 없는 곳이며, 저 또한 한번도 가본적이 없는 곳입니다. 그렇다면, 개인적으로 생각하시기에, 연우가 한 행위가 얼만큼 좋거나 나쁘다고 생각하십니까? 이 척도를 가리키며 말씀해주십시오. [참여자들에게 심각성 판단 척도를 보여주고, 아래에 응답을 기록하시오.]  [응답:] _________ [참여자가 가리킨 척도의 숫자를 기록하시오; 만약 참여자가 척도의 숫자들이 아닌 숫자와 숫자 사이 어딘가를 가리켰다면, 그것에서 가장 가까운 숫자를 적으시오.] |
| --- | --- |

| [권위자에의 의존] | 만약에 어떤 공동체의 리더들이 영향력 있는 사람이 뇌물을 받고 피의자를 변호하는 게 나쁘지 않다고 했다고 해봅시다. 공동체의 리더들이 그렇게 말했다면, 개인적으로 생각하시기에, 연우가 한 행위가 얼만큼 좋거나 나쁘다고 생각하십니까? 이 척도를 가리키며 말씀해주십시오. [참여자들에게 심각성 판단 척도를 보여주고, 아래에 응답을 기록하시오.]  [응답:] _________ [참여자가 가리킨 척도의 숫자를 기록하시오; 만약 참여자가 척도의 숫자들이 아닌 숫자와 숫자 사이 어딘가를 가리켰다면, 그것에서 가장 가까운 숫자를 적으시오.] |
| --- | --- |

**귀인 연구**

**[참가자들을 위한 서두]**

이제, 제가 방금 전에 들으셨던 시나리오들을 상기시켜드리고, 각각의 이야기에 대한 몇가지 질문들을 추가적으로 하겠습니다

# [#1: 외국인의 돈을 절도]

민준은 우리나라 다른 지방의 사람입니다. 근교에서 캠핑을 하다가 아이슬란드에서 온 외국인을 만났습니다. 그 외국인은 한국말을 할 줄 모릅니다. 외국인이 민준을 지나치고 나서, 가방을 내려놓고, 하천에서 손과 발을 씻기 위해 작은 언덕을 걸어 내려갔습니다. 그가 사라지고 나서, 민준은 그의 가방을 열고, 내용물을 봤습니다. 민준은 70만원을 발견했고, 돈을 가지고 빨리 그 자리를 떠나버렸습니다. 외국인은 아이슬란드로 돌아가기 전까지 자신의 돈을 누군가 가져갔다는 것을 알아차리지 못했고, 무언가를 하기에는 이미 늦었었습니다.

이제, 돈이 절도되게끔 한 가능한 상황의 모든 요인들을 잠시 생각해보십시오. 예를 들어, 민준이 돈을 훔치고 싶게 만든 그의 인생의 최근 사건들에 대해 생각해보십시오. 그리고, 그 외국인이 가방을 내려놓고 자리를 떠났었다는 사실도 고려해보십시오. 몇 초 동안 설문을 멈추고, 돈이 절도되게끔 한 모든 이유들을 생각해보시는 시간을 가지겠습니다.

[10초간 멈추시오]

- [**질문1 사람(person)**] 이제, 참여자분께서는 얼만큼이나, 다른 모든 이유들에 비해, 민준이 ‘어떤 종류의 사람’이기 때문에 돈이 절도되었다고 느끼시나요?

이 척도를 보시고, 답변해주세요. (*Person Scale*을 보여준다): [응답:] _________

- [**질문2 아무나(anyone)**] 이제, 참여자분께서는 얼만큼이나, (민준이 아닌) 다른 사람들이, 이와 동일한 상황에 놓여있을 때, 돈을 절도했을거라 느끼시나요?

이 척도를 보시고, 답변해주세요. (*Anyone Scale*을 보여준다): [응답:] _________

# 이제 제가 다른 상황을 상기해드리겠습니다.

# [#2: 구타]

이 일의 배경은 서울입니다. 준서는 그의 소지품들이 폭풍우 때문에 망가졌다는 것을 알고 집에 매우 화난 채로 돌아왔습니다. 준서는 아내가 소지품들이 망가진 것에 아무런 책임이 없다는 것을 알았음에도 불구하고 아내가 따뜻하게 인사하며 그를 맞이했을 때 단지 화가 났다는 이유만으로 아내의 얼굴을 세게 때렸고, 아내의 코에서 코피가 흘렀습니다.

이제, 아내가 맞게 되게끔 한 가능한 상황의 모든 요인들을 잠시 생각해보십시오. 예를 들어, 준서가 아내를 때리고 싶게 만든 그의 인생의 최근 사건들에 대해 생각해보십시오. 그리고, 폭풍우가 준서의 소지품들을 망가뜨렸다는 사실도 고려해보십시오. 몇 초 동안 설문을 멈추고, 아내가 맞게되게끔 한 모든 이유들을 생각해 보시는 시간을 가지겠습니다.

[10초간 멈추시오]

- [**질문1 아무나(anyone)**] 이제, 참여자분께서는 얼만큼이나, (준서가 아닌) 다른 사람들이, 이와 동일한 상황에 놓여있을 때, 아내를 때릴 거라 느끼시나요?

이 척도를 보시고, 답변해주세요. (*Anyone Scale*을 보여준다): [응답:] _________

- [**질문2 사람(person)**] 이제, 참여자분께서는 얼만큼이나, 다른 모든 이유들에 비해, 준서가 ‘어떤 종류의 사람’이기 때문에 아내가 맞게 되었다고 느끼시나요?

이 척도를 보시고, 답변해주세요. (*Person Scale*을 보여준다): [응답:] _________

# 이제 제가 다른 상황을 상기해드리겠습니다.

# [#3: 의도하지 않은 피해]

이 일의 배경도 역시 서울입니다. 현준과 승민은 진흙투성이인 길을 따라서 걷고 있었습니다. 현준은 진흙 속에서 미끄러졌고, 다시 균형을 잡는 와중에 의도치 않게 승민을 쳤습니다. 승민이 넘어지면서 팔을 다치게 되었습니다. 승민은 현준이 의도한 게 아니라는 걸 알고 있었습니다. 그럼에도 굉장히 화가 나서 승민이 일어나고서는 현준의 얼굴을 때렸습니다.

이제, 현준이 얼굴을 맞게 되게끔 한 가능한 상황의 모든 요인들을 잠시 생각해보십시오. 예를 들어, 승민이 현준을 때리고 싶게 만든 그의 인생의 최근 사건들에 대해 생각해보십시오. 그리고, 현준이 미끄러져서 승민이 넘어지게끔 했다는 사실도 고려해보십시오. 몇 초 동안 설문을 멈추고, 현준이 얼굴을 맞게 되게끔 한 모든 이유들을 생각해 보시는 시간을 가지겠습니다.

[10초간 멈추시오]

- [**질문1 사람(person)**] 이제, 참여자분께서는 얼만큼이나, 다른 모든 이유들에 비해, 승민이 ‘어떤 종류의 사람’이기 때문에 현준이 얼굴을 맞게 되었다고 느끼시나요?

이 척도를 보시고, 답변해주세요. (*Person Scale*을 보여준다): [응답:] _________

- [**질문2 아무나(anyone)**] 이제, 참여자분께서는 얼만큼이나, (승민이 아닌) 다른 사람들이, 이와 동일한 상황에 놓여있을 때, 현준을 때렸을거라 느끼시나요?

이 척도를 보시고, 답변해주세요. (*Anyone Scale*을 보여준다): [응답:] _________

# 이제 제가 다른 상황을 상기해드리겠습니다.

# [#4: 시장에서의 사기]

이 일의 배경도 역시 서울입니다. 어느 날 어떤 외국인이 광장시장에 음식을 사러 갔습니다. 그 외국인은 우리나라 사람이 아니고, 한국말을 할 줄 모릅니다. 그는 우리나라에서 굉장히 먼 핀란드에서 왔습니다. 외국인은 민성에게서 신선한 음식을 샀고, 음식에 대한 값을 지불했습니다. 하지만, 민성은 외국인에게, 그가 선택한 신선한 음식을 주지 않았습니다. 그가 고른 신선한 음식 대신 썩은 냄새가 나려고 하는 옛날 음식을 줬습니다. 민성은 외국인이 그 자리를 떠날 때까지 그 사실을 모르게 하기 위해서 음식을 잘 포장했습니다.

이제, 음식이 바뀌게 되게끔 한 가능한 상황의 모든 요인들을 잠시 생각해보십시오. 예를 들어, 민성이 음식을 바꿔서 주고 싶게 만든 그의 인생의 최근 사건들에 대해 생각해보십시오. 그리고, 외국인이 음식이 포장되는 동안 그것을 보고있지 않았다는 사실도 고려해보십시오. 몇 초 동안 설문을 멈추고, 음식이 바뀌게 되게끔 한 모든 이유들을 생각해 보시는 시간을 가지겠습니다.

[10초간 멈추시오]

- [**질문1 아무나(anyone)**] 이제, 참여자분께서는 얼만큼이나, (민성이 아닌) 다른 사람들이, 이와 동일한 상황에 놓여있을 때, 음식을 바꿀거라 느끼시나요?

이 척도를 보시고, 답변해주세요. (*Anyone Scale*을 보여준다): [응답:] _________

- [**질문2 사람(person)**] 이제, 참여자분께서는 얼만큼이나, 다른 모든 이유들에 비해, 민성이 ‘어떤 종류의 사람’이기 때문에 음식이 바뀌게 되었다고 느끼시나요?

이 척도를 보시고, 답변해주세요. (*Person Scale*을 보여준다): [응답:] _________

# 이제 제가 다른 상황을 상기해드리겠습니다.

# [#5: 명예훼손]

이 일의 배경도 역시 서울입니다. 동현과 성민은 이웃입니다. 하지만, 그들은 서로 좋아하지 않습니다. 어느 날, 동현은, 성민이 다른 사람의 물건 또는 돈을 훔쳤다는 거짓 소문을 퍼뜨렸습니다. 그 소문은 사실이 아님에도 불구하고, 많은 사람들은 그것을 믿었고, 성민은 자신의 평판에 치명적인 해를 입었습니다.

이제, 루머가 퍼지게 되게끔 한 가능한 상황의 모든 요인들을 잠시 생각해보십시오. 예를 들어, 동현이 루머를 퍼뜨리고 싶게 만든 그의 인생의 최근 사건들에 대해 생각해보십시오. 그리고, 동현과 성민이 둘다 서로를 좋아하지 않았다는 사실도 고려해보십시오. 몇 초 동안 설문을 멈추고, 루머가 퍼지게 되게끔 한 모든 이유들을 생각해 보시는 시간을 가지겠습니다.

[10초간 멈추시오]

- [**질문1 사람(person)**] 이제, 참여자분께서는 얼만큼이나, 다른 모든 이유들에 비해, 동현이 ‘어떤 종류의 사람’이기 때문에 루머가 퍼지게 되었다고 느끼시나요?

이 척도를 보시고, 답변해주세요. (*Person Scale*을 보여준다): [응답:] _________

- [**질문2 아무나(anyone)**] 이제, 참여자분께서는 얼만큼이나, (동현이 아닌) 다른 사람들이, 이와 동일한 상황에 놓여있을 때, 루머를 퍼뜨렸을 거라 느끼시나요?

이 척도를 보시고, 답변해주세요. (*Anyone Scale*을 보여준다): [응답:] _________

# 이제 제가 다른 상황을 상기해드리겠습니다.

# [#6: 부정의]

규태와 태경은 서울출신의 청년들입니다. 어느 날 밤에 그 둘은 언쟁을 했습니다. 규태가 먼저 태경과 싸움을 시작했고, 그에게 부상을 입혔습니다. 며칠이 지난 뒤에, 그 사건에 대해서 어떤 조치가 취해져야 하는지 논의하는 지역사회의 회의가 있었습니다. 회의 전에, 서울에서 영향력 있는 인물인 연우는 규태로부터 뇌물로 돈을 받았습니다. 연우는 회의에서 거짓말을 했고, 태경이 싸움을 시작했다고 말했습니다. 모두가 연우를 믿었고, 지역사회에서 규태가 아닌 태경을 처벌하기로 했습니다.

이제, 누가 싸움을 시작했는지에 대한 거짓말이 나오게끔 한 가능한 상황의 모든 요인들을 잠시 생각해보십시오. 예를 들어, 연우가 거짓말을 하는 대가로 뇌물을 받고 싶게 만든 그의 인생의 최근 사건들에 대해 생각해보십시오. 그리고, 왜 규태와 태경이 언쟁을 했는지 가능한 이유들에 대해서 고려해보십시오. 몇 초 동안 설문을 멈추고, 누가 싸움을 시작했는지에 대한 거짓말이 나오게 되게끔 한 모든 이유들을 생각해 보시는 시간을 가지겠습니다.

[10초간 멈추시오]

- [**질문1 아무나(anyone)**] 이제, 참여자분께서는 얼만큼이나, (연우가 아닌) 다른 사람들이, 이와 동일한 상황에 놓여있을 때, 누가 싸움을 시작했는지에 대한 거짓말을 할거라 느끼시나요?

이 척도를 보시고, 답변해주세요. (*Anyone Scale*을 보여준다): [응답:] _________

- [**질문2 사람(person)**] 이제, 참여자분께서는 얼만큼이나, 다른 모든 이유들에 비해, 연우가 ‘어떤 종류의 사람’이기 때문에, 누가 싸움을 시작했는지에 대한 거짓말이 나오게 되었다고 느끼시나요?

이 척도를 보시고, 답변해주세요. (*Person Scale*을 보여준다): [응답:] _________

감사합니다, 이제 설문을 모두 마치셨습니다. 이제, 참여자분에 대한 질문 몇가지만 더 하겠습니다.

[인구통계 질문으로 넘어가십시오.]

# [인구 통계 자료]

이 설문에 참여해주셔서 감사합니다. 참여자 분에 대한 추가적인 질문을 하겠습니다. 만약 밝히길 원하지 않는 항목이 있다면 작성하지 않으셔도 좋습니다.

*성별*: ________ *나이:* ________ *인종:* _______________ *태어난 나라:* ____________

*현재국적:*

- 한국
- 다른 나라
- 노출하지 않길 원함

*학력:* _____________________________

*부모님의 학력:* ______________________________

*연 가계 소득:* ______________________________

*정치적인 입장을 묘사하자면 다음 중 어떤 것입니까?*

- 매우 진보적
- 약간 진보적
- 중도
- 약간 보수적
- 매우 보수적

기타 덧붙이실 것 (방해되는 것이나, 신경 쓰이는 점이 있으셨는지, 이해가 어렵지 않았는지, 성실하게, 정직하게 답변했는지, 등등):

______________________________________________________________________________________________________________________________________________________________________________________________________________________________________________________________________________________________________________________________________________________________________________________________________

이 연구에 참여해주셔서 감사합니다. 이 연구를 만든 연구자들은 사람들의 옳고 그름에 대한 판단과 이런 판단들이 행위가 일어난 장소, 시간과 같은 맥락에 영향을 받는지에 대해 배우는 것에 관심이 있습니다. 다시 한번 감사드립니다.

[참여자에게 돈을 지급함.]
